# Artificial intelligence in ovarian cancer: advancing in precision diagnosis and clinical management

**DOI:** 10.3389/fimmu.2026.1795144

**Published:** 2026-05-07

**Authors:** Mingjun Shao, Tong Wang, Limei Ji, Lili Xu, Yanfei Zhang, Dongge Wang, Cenlin Jia, Lin Chen, Heng Zhang, Wei Yan, Xuehao Cui, Ran Tong

**Affiliations:** 1 Affiliated Jinhua Hospital, Zhejiang University School of Medicine, Jinhua, Zhejiang, China; 2 Jinhua Maternity and Child Health Care Hospital, Jinhua, Zhejiang, China; 3 Department of Biology, Duke University, Durham, NC, United States; 4 Department of Neurosurgery, The Second Affiliated Hospital, Zhejiang University School of Medicine, Hangzhou, China; 5 Department of Clinical Neuroscience, University of Cambridge, Cambridge, United Kingdom; 6 Mathematics and Statistics Department, University of Texas at Dallas, Richardson, TX, United States

**Keywords:** artificial intelligence, electronic health records, imagine-based, multimodal data integration, ovarian cancer, prognostic prediction

## Abstract

**Background:**

Ovarian cancer remains one of the deadliest gynecologic malignancies. Poor outcomes largely reflect late diagnosis, marked inter- and intratumoral heterogeneity, and variable treatment response.

**Methods:**

This review summarizes recent advances in artificial intelligence (AI) for ovarian cancer research and clinical care, focusing on imagine-based radiology, digital pathology; longitudinal clinical data/Electronic Health Record (EHR), and spatial-temporal multi-omics.

**Results:**

AI approaches have been applied to tumor detection and classification, prognostic risk stratification, and treatment response prediction. Multimodal models that integrate imaging, molecular profiling, and clinical data enable more refined characterization of tumor heterogeneity and the tumor microenvironment, supporting improved diagnosis, risk assessment, and individualized management.

## 1 Introduction

Despite therapeutic advances, ovarian cancer remains a leading cause of death in gynecologic oncology (1). High-Grade Serous Ovarian Carcinoma (HGSOC) is the most aggressive histologic subtype (2). A significant proportion of patients are diagnosed at advanced stages, correlating with unfavorable survival results. The Cancer Genome Atlas (TCGA) has indicated that HGSOC is characterized by almost universal Tumor Protein p53 (TP53) mutations. These molecular traits explain why different malignancies respond differently to certain therapies (3). These molecular characteristics lead to early treatment resistance and recurrent relapses, which continue to pose significant therapeutic hurdles.

Current management is constrained by delayed diagnosis and variable therapy response. Histopathological assessment of hematoxylin-eosin (H&E) staining and immunohistochemistry (IHC) constitutes the diagnostic gold standard; nonetheless, it is time-intensive and prone to inter-observer variability (4). Radiologic modalities, including computed tomography (CT), magnetic resonance imaging (MRI), and ultrasound, are crucial for detection and staging; nevertheless, conventional interpretation may overlook nuanced imaging correlates of tumor biology (5). Commonly utilized biomarkers exhibit restricted sensitivity and specificity for early-stage illness and for forecasting therapy response (6). These limitations underscore a significant obstacle in the therapy of ovarian cancer: the growing number and complexity of clinical, imaging, and genetic data are challenging to handle with traditional analytical methods.

In this instance, AI, particularly machine learning (ML) and deep learning (DL), can model complex patterns in high-dimensional clinical, imaging, and molecular data, and enable automated complicated connections between large groups of patients (7). Research indicates that image-based analysis and digital pathology can attain performance levels equivalent to those of human experts. In ovarian cancer, first trials integrating imaging results with fundamental clinical data have already shown significant advantages (8). However, limited transparency, domain shift, and bias remain key barriers still make AI challenging in regular clinical practice (9).

**Figure 1:**
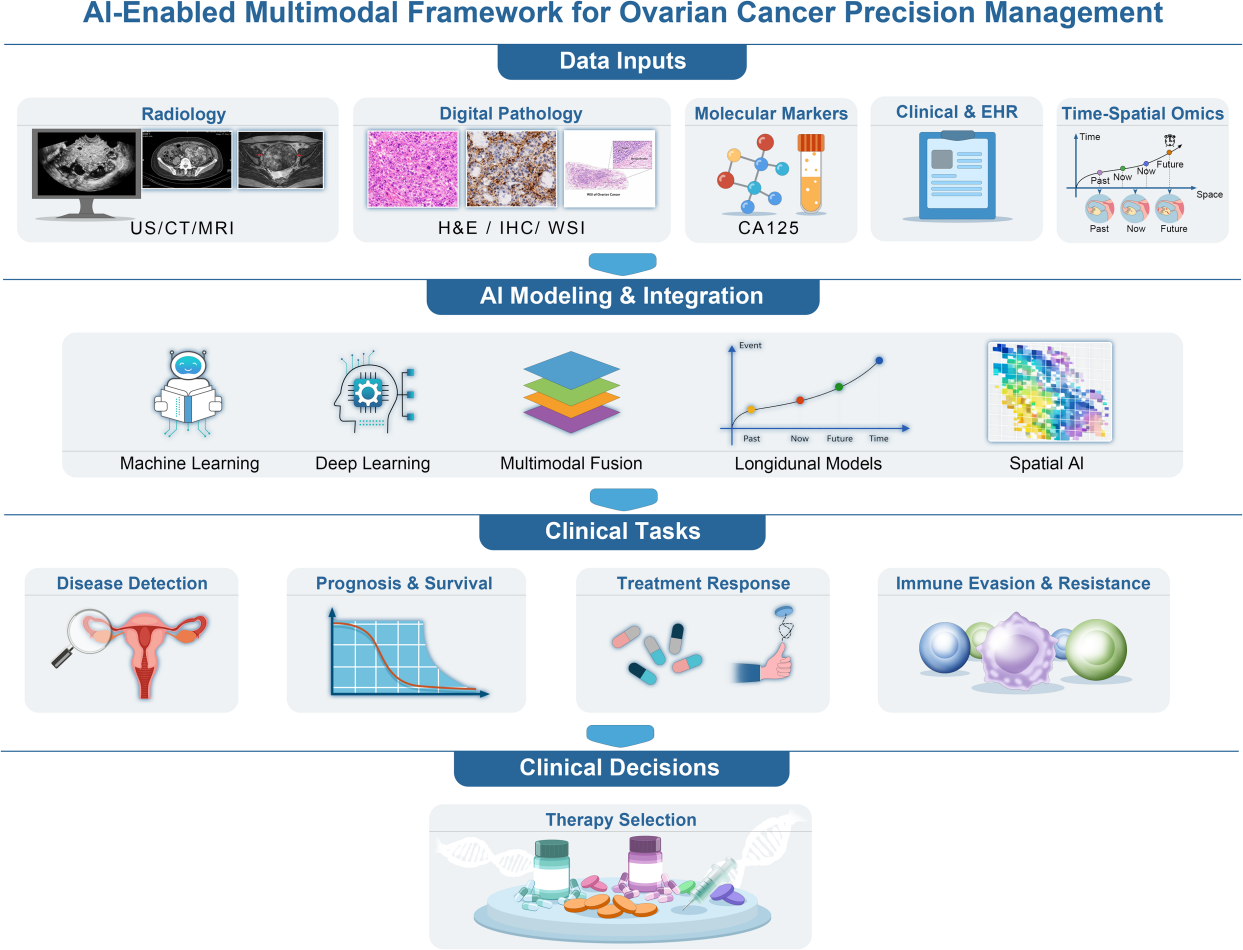
AI-enabled multimodal framework for precision management of ovarian cancer. An integrated framework using AI to facilitate precision management of ovarian cancer. This framework combines multiple data modalities, including radiology (ultrasound, CT, MRI), digital pathology (H&E staining, immunohistochemistry, whole-slide images), molecular profiling (e.g., CA125), clinical data, EHR data, and spatial-temporal multi-omics. AI models process these diverse data sources for early detection, risk stratification, treatment response prediction, immune evasion, and personalized therapeutic decision-making. By integrating imaging, clinical, and molecular data, the framework enhances ovarian cancer diagnosis, prognosis, and treatment selection, leading to more accurate and personalized care.

Accordingly, this review synthesizes progress across six principal domains (1) Early Detection and Treatment Response Prediction with imaging and pathology; (2) Prediction of HRD from Routine Pathology; (3) Clinical Data Integration using longitudinal records; (4) Multimodal Data Fusion strategies; (5) Spatial Omics and AI for mapping tumor ecosystems; and (6) Model Interpretability and Clinical Translation considerations. In general, ovarian cancer AI is moving away from performance-based models and toward clinically useful systems. This review shows how AI research can be in line with real-world decision-making (**Figure 1**).

## 2 Image-based detection and treatment response prediction

Image-based AI techniques mainly facilitate early diagnosis, risk stratification, and clinical triage in ovarian cancer.

### 2.1 Radiology for detection

The two main categories of contemporary approaches are radiomics and DL models. Radiomics uses hand-crafted quantitative imaging features like shape and texture, whereas DL models automatically learn discriminative patterns straight from raw images (10). Ultrasound is still the best way for evaluating adnexal masses, and accumulating evidence shows that AI can make it much easier to tell the difference between benign and malignant ovarian lesions. A meta-analysis of histopathology-validated studies reported pooled sensitivity ~81% and specificity ~92% across >15, 000 ultrasound images, supporting clinical utility while underscoring the need for prospective validation (11).

These findings enhance the diagnostic efficacy of AI and emphasize the need for further clinical validation. AI performance involves a trade-off between sensitivity and specificity. High sensitivity is crucial for early detection but can increase false positives. While a high AUC suggests good performance, it doesn’t guarantee the model is ready for primary screening. Optimizing for sensitivity alone could lead to unnecessary treatments, underscoring the importance of balancing sensitivity and specificity in clinical use.

Residual Network with Feature Pyramid Network (ResNet-FPN) based Convolutional Neural Networks (CNNs) have been used for CT-based tumor classification by capturing multi-scale features relevant to lesion size, shape, and texture (12). Three-dimensional (3D) DL architectures go beyond slice-based analysis by using volumetric Positron Emission Tomography (PET) data. This makes it possible to estimate tumor load and disease stage more accurately. Models based on ResNet that are tailored for 3D input have shown better performance in diagnosing and staging, and attention-based visualization supports their clinical relevance (13). Subgroup studies demonstrated that AI models utilized in MRI attained diagnostic performance on par with ultrasound- and CT-based methodologies, highlighting the efficacy of MRI-based AI in ovarian cancer diagnosis (14). AI-enhanced ADNEX model (ADNEX-AI) was made for a multi-task DL system that automates feature extraction within the International Ovarian Tumor Analysis (IOTA) Assessment of Different NEoplasias in the adneXa (IOTA-ADNEX) ultrasonography framework that is approved by the guidelines. In a large, multicenter study, ADNEX-AI was able to tell the difference between benign and malignant ovarian masses (AUC ≈ 0.93) by automatically separating important tumor parts (lesion, locules, solid tissue, and papillary projections) and measuring their attributes (15). Importantly, the AI model’s performance was similar to that of expert-derived Assessment of Different NEoplasias in the adneXa (ADNEX) ratings, but it had better calibration and less variability between centers. These results suggest that such approaches could support ovarian mass triage in settings with limited specialist ultrasound expertise. In a multicenter study of 17, 119 images from 3, 652 patients across 20 centers in eight countries, performance matched or exceeded expert assessment and could reduce specialist referrals by up to 63% (16). These DL architectures, such as ResNet-FPN and 3D CNNs, automatically learn hierarchical spatial features, ranging from low-level texture and edge patterns to high-level structural cues, through stacked convolutional filters and multi-scale feature integration. This enables them to detect subtle lesion characteristics, such as fine texture differences and shape irregularities, that are difficult for handcrafted methods and may be overlooked by human observers (17, 18).

Integrating imaging with clinical variables generally improves risk stratification compared with clinical data alone (19).

### 2.2 Digital pathology and prognosis

Pathologists diagnose ovarian endometrioid carcinoma by examining Hematoxylin and eosin (H&E) slides, where it typically appears as dense glandular clusters with back-to-back arranged glands, showing brick-like or sieve/maze-like patterns, consisting of tall columnar epithelial cells with abundant cytoplasm and oval-shaped nuclei in a pseudostratified arrangement (20). H&E staining is the gold standard for diagnosing ovarian cancer, and is the primary imaging input used in digital pathology and AI/ML models (21). These pathological features, including glandular structure, nuclear morphology, necrosis, and invasion patterns, are key diagnostic criteria for ovarian endometrioid carcinoma. Emphasizing the features pathologists focus on in H&E staining and how AI models use them can enhance clinician trust in AI predictions.

Existing studies have used DL to segment tissue/cells in whole-slide images, automatically quantifying the shape, texture, and number of cells and nuclei. These features, such as nuclear size, shape, staining intensity, glandular distribution, and density, can be used to train classifiers to distinguish tumor types or predict molecular characteristics (22, 23). AI-driven digital pathology enables fine-grained tissue-level analysis, bridging morphological patterns with molecular and clinical outcomes. In ovarian cancer, H&E whole-slide image (WSI) provide rich morphological information for classification and prognostic modeling (24).

Using AI-based analysis of digital pathology slides improved diagnosis and prognosis assessment in gynecologic cancers (25). New imaging analysis methods have helped improve ovarian cancer detection and care, although several challenges still limit their routine use in clinical practice (26). Recent research has methodically evaluated the efficacy of five pre-trained CNNs (VGG16, VGG19, ResNet50, MobileNet, and DenseNet121) for the classification of ovarian cancer utilizing histopathology images, in comparison to traditional ML classifiers. DenseNet variants often performing best in histopathology-based classification (27).

Homologous Recombination Repair (HRR) is a high-fidelity repair pathway for double-strand DNA breaks. Dysfunction in HRR forces tumor cells to rely on error-prone repair mechanisms, leading to genomic instability and the accumulation of hallmark features, such as Loss of Heterozygosity (LOH), Telomeric Allelic Imbalance (TAI), and Large-Scale Transitions (LST) within the genome (28, 29). Homologous recombination deficiency (HRD) is a significant clinical indicator in ovarian cancer. It most often arises from mutations in BRCA1 or BRCA2, or from alterations in other DNA repair genes, and is associated with increased sensitivity to platinum-based chemotherapy and Poly (ADP-ribose) Polymerase (PARP) inhibitor treatment (30). HRD, initially characterized by BRCA1/2 mutations, underlies the clinical efficacy of PARP inhibitors in ovarian cancer and serves as a fundamental diagnostic for broadening precision medicine beyond BRCA-mutant illness in ovarian cancer (31). Approximately 25% of ovarian cancer patients harbor germline BRCA mutations, the majority of which result in HRD, while an additional 5-7% exhibit somatic HRD (32). Data from TCGA indicate that nearly half of HGSOC display defects in homologous recombination repair (33). The HRD status is closely related to the efficacy of PARP inhibitors. HRD-positive patients receiving PARP inhibitors achieve Longer Progression-free Survival (PFS) and survival benefits.

DeepHRD and other DL models may directly determine homologous recombination failure from standard H&E-stained pathology slides and have demonstrated strong performance in separate cancer groups. AI-predicted HRD is significantly linked to better results in patients treated with platinum, underscoring histology-based AI as a scalable alternative for HRD evaluation in precision oncology (34). Patient-level DL models like HRDPath show that HRD can be reliably predicted from routine histopathology in HGSOC, making it a digital biomarker that can be used to guide therapy (35).

Yang et al. have developed graph-based DL models using H&E-stained WSIs to predict prognosis and therapeutic response in ovarian cancer. The Ovarian Cancer Digital Pathology Index (OCDPI), trained on TCGA data and validated in independent multicenter cohorts, demonstrated robust prognostic value for overall survival and recurrence risk, independent of established clinicopathological factors (36).

**Figure 2:**
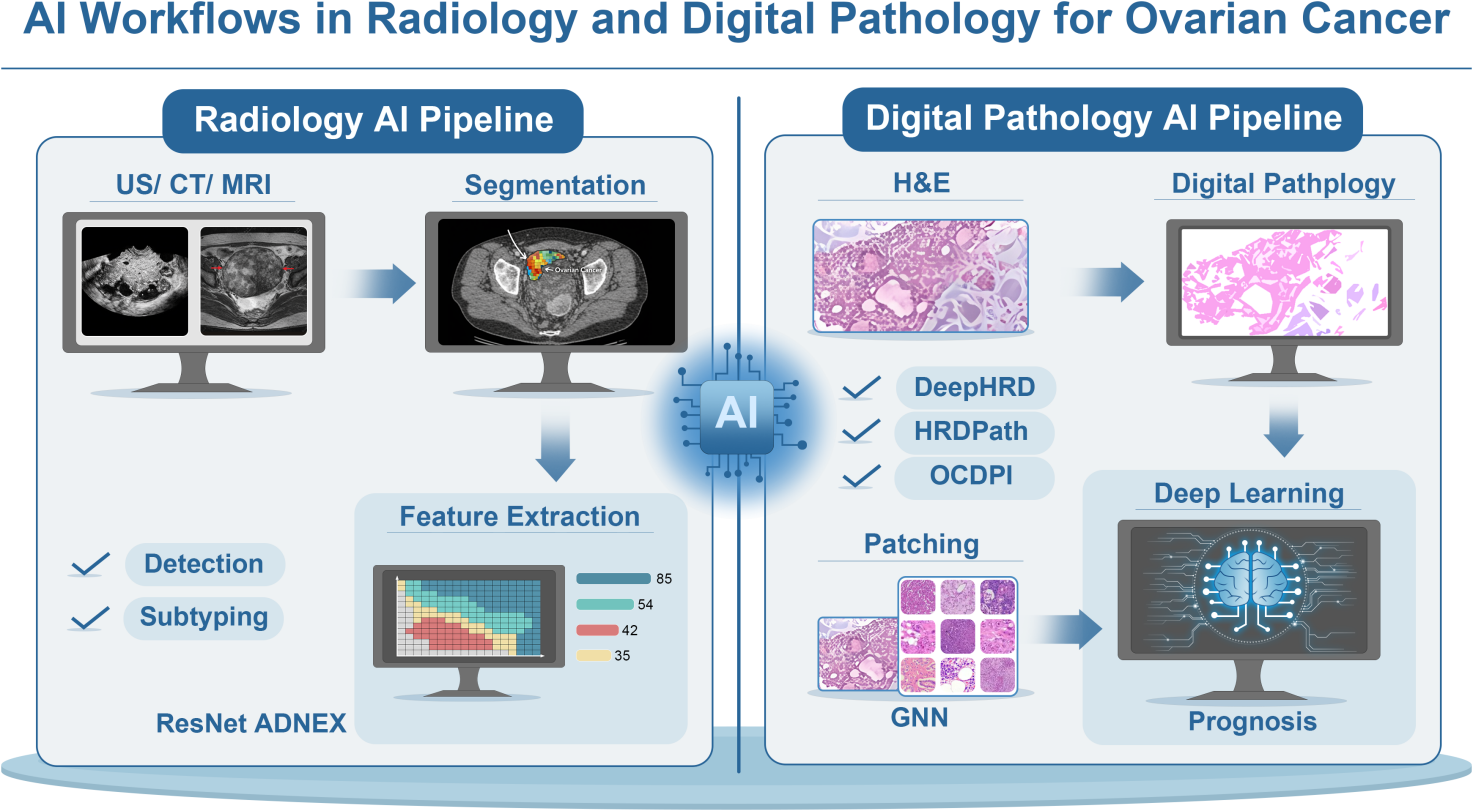
AI workflows in radiology and digital pathology for ovarian cancer. AI workflows in radiology and digital pathology used for ovarian cancer detection and prognosis. In radiology, AI techniques like radiomics and DL models analyze imaging data (ultrasound, CT, MRI) to detect, classify, and stage tumors. Tasks include lesion segmentation, feature extraction (such as shape, texture, and size), and lesion subtyping, with models such as ResNet ADNEX applied to classify ovarian masses. In digital pathology, AI models process WSI from H&E-stained tissue sections, segmenting and analyzing tissue morphology (e.g., glandular structure, nuclear morphology, necrosis). These AI workflows improve diagnostic accuracy, support risk stratification, and assist in clinical decision-making regarding therapy.

Overall, imaging- and pathology-based AI supports early detection, subtype characterization, and prognostic risk stratification, although standardization and external validation remain essential for clinical deployment (**Figure 2**).

**Table 1** provides an overview of the major data modalities in ovarian cancer and the AI approaches and tasks for which they have been employed.

**Table 1:** Representative image-based AI studies for ovarian tumor detection.

ML/DL	Device	Algorithm Architecture	Performance	Author (year)
ML	Ultrasound	PNN	10-fold cross-validation, reaching an accuracy of 99.8%, sensitivity of 99.2%, and specificity of 99.6%.	Acharya et al., 2014 (37)
ML	Ultrasound	Logistic Regression (LR), NN	170 operated adnexal masses (18% malignant), externally models variable performance (AUC 0.69-0.90), with maximum AUC of 0.86; at near-perfect sensitivity, specificity was limited (0.45-0.60).	Mol et al., 2019 (38)
ML	Ultrasound	k-Nearest Neighbors (KNN), LD, Support Vector Machine (SVM), ELM	KNN showed poor performance (accuracy < 60%), whereas LD-, SVM-, and ELM-based classifiers achieved robust accuracy (>85%).	Martinez et al., 2019 (39)
ML	Ultrasound	SVM, KNN, RF, NB, XGBoost	XGBoost achieved the highest accuracy (0.80), followed by RF (0.78), logistic regression (0.67), and SVM (0.62).	Akazawa et al., 2020 (40)
DL	Ultrasound	DCNN	ResNet34 showed strong performance (AUC 0.91-0.96)	Wang et al., 2021 (41)
ML	Ultrasound	SVM	Accuracies of 80%-87%, sensitivities of 75%-81%, and AUCs of 0.87-0.89 across mass types.	Chiappa et al., 2021 (42)
ML	Ultrasound	KNN	Outperformed KNN in distinguishing benign from malignant lesions (sensitivity 90.5% vs 71.4%-80.0%; specificity 93.1% vs 87.5%-89.8%).	Stefan et al., 2021 (43)
DL	Ultrasound	Deep Neural Network (DNN)	Ovry-Dx1: sensitivity 96.0%, specificity 86.7% (SA 88.0%, P = 1.0); Ovry-Dx2: sensitivity 97.1%, specificity 93.7% (12.7% inconclusive); combined approach: sensitivity 96.0%, specificity 89.3% (P = 1.0).	Christiansen et al., 2021 (44)
ML	Ultrasound	SVM	Accuracies increasing from 75%-85% to 86%-90% after feature fusion.	Al-Karawi et al., 2021 (45)
DL	Ultrasound	DCNN	Deep Convolutional Neural Network (DCNN)-assisted radiologists achieved an average accuracy of 0.876 (p<0.05)	Gao et al., 2022 (46)
DL	Ultrasound	CNN	DLTVS: AUC 0.95 (95% Confidence Interval (CI) 0.93-0.97), comparable to DLTAS (AUC 0.95) and outperforming DLCDFI_TVS (AUC 0.88).	Miao et al., 2023 (47)
DL	Ultrasound	CNN	YOLOv8x showed superior precision (+19% vs YOLOv7) with GPU/CPU speeds of 186/1.84 fps	Pham and Le, 2024 (48)
DL	Ultrasound	CNN	ResNet-101 achieved an AUC of 0.93, accuracy of 84.9%, sensitivity of 93.5%, and specificity of 81.7%	Liu et al., 2024 (49)
DL	Ultrasound	Multi-task CNN	ADNEX-AI AUC 0.930 (95% CI 0.913-0.943), slightly lower but comparable to examiner-derived ADNEX (AUC 0.945; 95% CI 0.930-0.957; P = 0.004).	Geysels et al., 2025 (15)
DL	Ultrasound	Transformer-based neural networks	17, 119 images (3, 652 patients; 20 centers; 8 countries); leave-one-center-out validation; superior to experts across F1, sensitivity, specificity, accuracy, κ, MCC, DOR, Youden’s J; 63% reduction in expert referrals.	Christiansen et al., 2025 (16)
ML	CT, MRI	RF	Type I and type II EOCs with moderate accuracy (AUC = 0.808; accuracy = 73.5%),	Liu D et al., 2017 (50)
ML	CT	LR	AUC = 0.854; sensitivity = 78.8%; specificity = 90.7%), outperforming radiomics- or clinical-only models	Hu et al., 2021 (51)
ML	CT	SVM	AUCs of 0.73-0.86 (accuracy 0.69-0.78) for distinguishing Serous Borderline Ovarian Tumors (SBOTs) from Seromucinous Ovarian Tumors (SMOTs), with comparable performance across AP, VP, and EP phases.	Yu et al., 2021 (52)
ML	CT	LR	2D model: AUC 0.96-0.97, validation accuracy 90.2%, sensitivity 100.0%, specificity 82.6%; 3D model: AUC 0.96-0.99, validation accuracy 97.6%, sensitivity 95.7%, specificity 100.0%; no significant difference between models (P > 0.05).	Li et al., 2022 (53)
DL	CT	CNN + Feature Pyramid Network	ResNet50V2-FPN + CT sequence selection:accuracy 0.89-0.90 (vs 0.80-0.82 without selection), precision 0.83-0.86, recall 0.96-1.00, F1 0.90	Bhuvaneshwari KV et al, 2024 (12)
DL	PET/CT	CNN	OCDA-Net achieved 92% accuracy for ovarian cancer detection and 94% accuracy for staging, outperforming ResNet, DenseNet, GoogLeNet, U-Net, VGG, and AlexNet.	Sadeghi MH et al., 2025 (13)
ML	MRI	SVM, LDA	Using TTP and WIR parameters, high performance (accuracy 89%, AUC 0.93), with sensitivities up to 89% (LDA) and 97% (SVM) and specificities up to 93% (LDA) and 100% (SVM).	Kazerooni et al., 2017 (54)
ML	MRI	Least Absolute Shrinkage and Selection Operator (LASSO)	Accuracies of 0.87-0.90 for benign vs malignant masses and 0.84-0.93 for type I vs type II subtypes; higher radiomics risk scores were associated with poorer prognosis (HR = 4.17, p = 0.001).	Zhang et al., 2019 (55)
DL	MRI	MAC-Net	AUC: 0.878 (MAC-Net)	Jian et al., 2021 (56)
DL	MRI	CNN	Ensemble model: accuracy 0.87, specificity 0.92, sensitivity 0.75; higher than junior (0.64/0.64) and senior radiologists (0.74/0.70). AI assistance increased junior radiologists’ accuracy to 0.77 and specificity to 0.81.	Wang et al., 2021 (57)
ML	MRI	LASSO	2D and 3D T2WI-based radiomics models achieved accuracies of 0.78-0.99.	Liu et al., 2022 (58)
ML	MRI	LR	MP-ST: AUC 0.932/0.902 (internal/external), better than MP-WT (0.917/0.767); BEOT vs early MEOT AUC 0.909-0.920; radiologists mean AUC ~0.79.	Li et al., 2020 (59)
ML	MRI	LASSO	Combined radiomics model outperformed single-parameter models, achieving AUCs of 0.806 (internal) and 0.847 (external)	Jian et al., 2020 (60)
DL	MRI	MICNN	Both EMP and LMP models differentiated Borderline Epithelial Ovarian Tumor (BEOT) from MEOT with AUCs of 0.855 and 0.884, respectively, and the LMP model outperformed radiologists (AUC 0.884 vs. 0.797)	Jian et al., 2022 (61)
ML	MRI	LASSO	Combined model AUC = 0.954, accuracy = 0.839, precision = 0.909), exceeding both the clinical model (AUC = 0.847) and the radiomics model (AUC = 0.807).	Zheng et al., 2022 (62)
DL	Histopathology	CNN	DenseNet121 showed best performance (accuracy 95.3%).	Bikku T et al., 2025 (27)
DL	Histopathology	CNN	DeepHRD’s AUC 0.76-0.81 with improved PFS (HR = 0.45) and OS (HR = 0.46-0.49).	Bergstrom EN et al., 2024 (34)
DL	Histopathology	Multi-model Transformer	HRDPath showed robust performance (AUC 0.846; specificity 0.938), exceeding previous H&E-based HRD models.	Wu CI et al., 2025 (35)
DL	Histopathology	CNN + Transformer	Effectively stratified overall survival (HR 1.9-2.8) across multicenter cohorts.	Yang et al., 2023 (36)

## 3 Clinical data integration for prognosis and risk stratification

**Figure 3:**
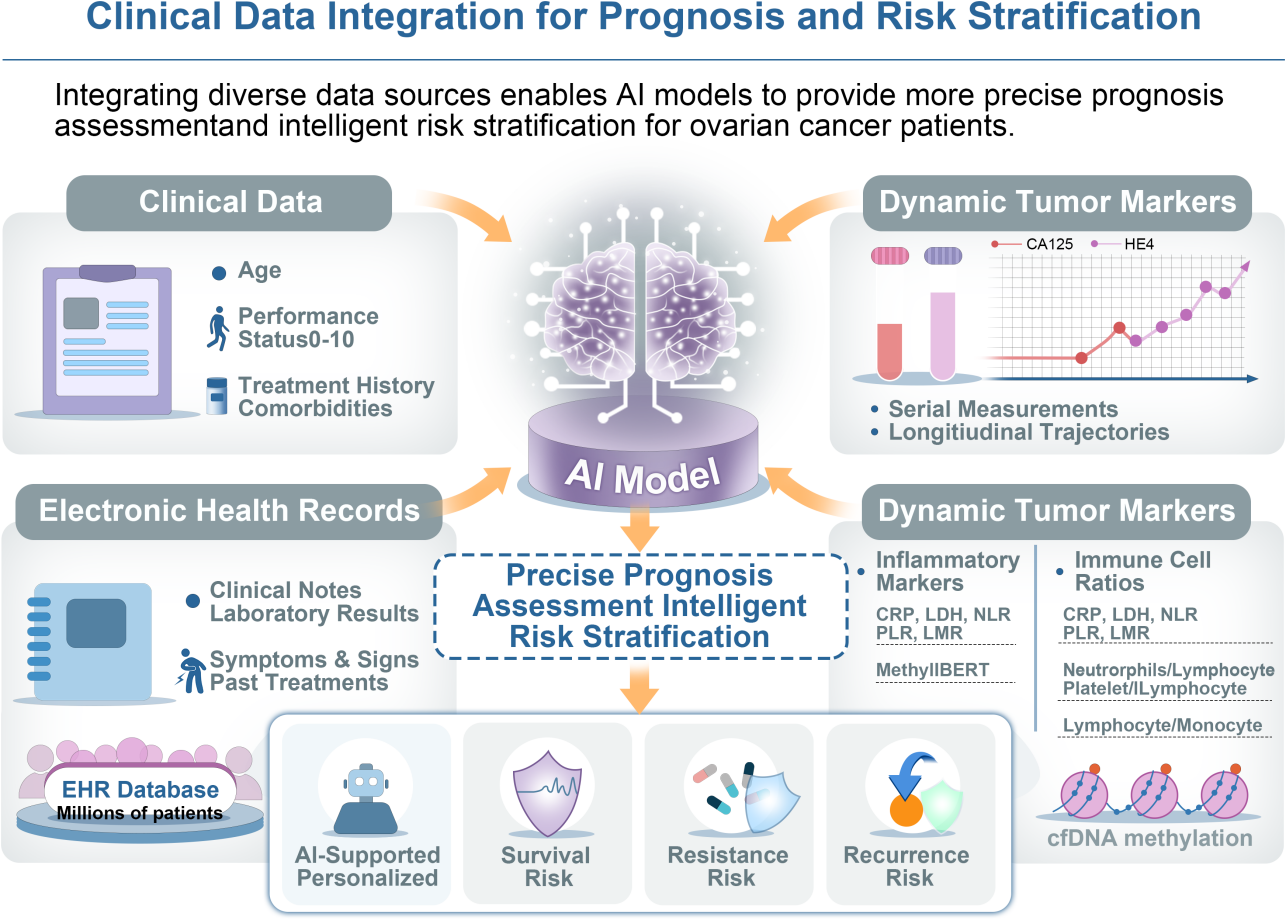
Clinical data integration for prognosis and risk stratification in ovarian cancer. Integration of clinical data for prognosis and risk stratification in ovarian cancer. AI models combine clinical information, including demographics, performance status, comorbidities, tumor markers (e.g., CA125, HE4), EHRs (including clinical notes, laboratory results, symptoms, and prior treatments), and dynamic inflammatory and immune-related markers are jointly incorporated into a unified model. By leveraging serial measurements and large-scale EHR data, the framework enables individualized prediction of survival risk, treatment resistance, and recurrence risk, supporting more precise and personalized clinical decision-making.

Clinical data integration underpins prognosis and risk stratification by capturing patient-level risk factors in ovarian cancer. In addition to images data, the longitudinal clinical record of an ovarian cancer patient harbors valuable information for prognosis and treatment personalization (**Figure 3**). Factors such as a patient’s age, performance status, prior treatment history, comorbidities, and dynamic tumor marker levels (e.g. serial Cancer Antigen 125 (CA125) measurements) all influence outcomes. For example, large prospective cohort studies such as the SOCFCP integrate clinical, genetic, lifestyle, and biospecimen data to enable biomarker discovery and predictive modeling for ovarian cancer diagnosis, treatment, and prognosis (63). Moreover, AI models are increasingly incorporating these real-world clinical data to improve predictive performance in ovarian cancer, moving beyond static snapshot predictions to a more holistic, longitudinal view of the patient (64).

### 3.1 Markers for diagnosis and prediction

Overall, routine markers remain central to risk modelling, and their value increases when combined with complementary clinical or imaging information.

Tumor markers are widely used as structured input features for ovarian cancer prediction because they provide quantitative signals related to tumor burden and malignancy. A large retrospective cohort compared multiple statistical and ML models for estimating malignancy risk across pathological categories. Using clinical and ultrasound features, with or without CA125, models showed consistently strong discrimination between benign and malignant tumors (Area Under the Receiver Operating Characteristic Curve (AUROC) ~0.89-0.92) (65). Although overall diagnostic accuracy was similar across approaches, tree-based ensemble models such as random forest (RF) and XGBoost showed better multiclass calibration and slightly higher clinical net benefit at commonly used risk thresholds and its importance has been consistently confirmed across algorithms such as RF, Recurrent Neural Networks (RNNs), and gradient boosting methods. RF, RNN, and Extreme Gradient Boosting (XGBoost), also achieved high accuracy when incorporating CA-125 (66). Models that combined CA125, HE4, and routine hematological features also achieved diagnostic accuracies above 85%, with CA125 and HE4 consistently ranking among the most informative variables (67). Gradient boosting models further supported the diagnostic contribution of HE4 and CA72–4 for distinguishing ovarian cancer from benign tumors. The longitudinal multivariable modeling approaches, including Bayesian change-point detection and RNN, integrated serial CA125 and HE4 measurements for early ovarian cancer detection (68). The significance of ML in ovarian cancer management lies in improving early detection accuracy, optimizing treatment planning, significantly surpassing traditional statistical methods, and promoting personalized treatment, ultimately improving patient prognosis (69).

Inflammatory markers reflect the association between systemic inflammation and cancer progression. For blood markers, AI-based models integrating circulating tumor cells and other blood-derived biomarkers achieved good performance in predicting platinum sensitivity and disease stage in ovarian cancer, with AUCs of approximately 0.80 (70). In Endometrioid Ovarian Carcinoma (EOC), blood-based AI models have been developed for preoperative diagnostic and prognostic prediction by integrating routine peripheral blood biomarkers. Using supervised ML approaches, particularly ensemble tree-based methods such as RF and gradient boosting, these models achieved high accuracy in distinguishing EOC from benign ovarian tumors (AUC up to 0.97), and showed moderate performance in predicting clinical stage, histologic subtype (including high-grade serous and mucinous tumors), residual tumor burden, and survival risk (71). A 2025 systematic review and meta-analysis evaluated the diagnostic performance of AI-derived blood biomarkers for ovarian cancer and demonstrated overall favorable accuracy. In 40 investigations, AI-based models had a pooled sensitivity of around 85% and a pooled specificity of about 91%. Their performance was particularly strong in analyses based on serum markers with external validation (72). These findings suggest that blood-based biomarkers could function as noninvasive tools for ovarian cancer diagnosis. Furthermore, inflammatory and hormonal indicators such as C-Reactive Protein (CRP), LDH, the Neutrophil-to-Lymphocyte Ratio (NLR), the Platelet-to-Lymphocyte Ratio (PLR), and the Lymphocyte-to-Monocyte Ratio (LMR), in addition to immune cell ratios, have been incorporated into ML models. This combination enhances the capacity to predict ovarian cancer by revealing inflammation and immunological dysregulation associated with malignancies (73, 74).

Epigenetic markers, including transformer-based analysis of cfDNA methylation, have shown considerable diagnostic precision. By identifying disease-specific methylation indicators and transforming them into Droplet Digital Polymerase Chain Reaction (ddPCR)-based assays, these approaches highlight a feasible approach for non-invasive and therapeutically relevant early screening (75).

When ultrasound results were paired with CA125 and HE4, the diagnosis was more accurate than when imaging was utilized alone. The integrated model exhibited significant accuracy and specificity, indicating that traditional biomarkers like CA125 and HE4 possess increased diagnostic utility when combined with imaging features (76). A multicenter retrospective study has created a combined prediction method that uses multi-criteria decision making-based classification fusion to merge information from different test findings to make diagnoses more accurate. The AI model reached an AUC of 0.949 in internal validation and steady performance in two external validation cohorts (AUCs ≈0.88). Using 51 routine laboratory markers and age, the model outperformed CA125 and HE4, particularly in early-stage ovarian cancer. Its performance remained robust even without key tumor markers, suggesting a low-cost and widely applicable approach for risk prediction (77). **Table 2** provides a structured overview of representative AI-based diagnostic studies leveraging diverse biomarkers in ovarian cancer.

**Table 2:** Representative biomarker cases of AI based ovarian cancer diagnosis.

Sample type	Algorithms	Biomarkers and features	Performance	Reference
349 Chinese patients with 49 variables	MRMR feature selection + Decision Tree	HE4 and CEA	DT: Acc = 95.6%, AUC = 0.949; Risk of Ovarian Malignancy Algorithm (ROMA): Acc = 92.1%, AUC = 0.943; LR: Acc = 97.4%, AUC = 0.969	M. Lu et al., 2020 (78)
Retrospective clinical cohort	SVM; Ensemble Subspace Discriminant; Logistic Regression; feature selection using exhaustive search, chi-square test, and MRMR	CA125	AUC: Ridge MLR 0.90; SVM 0.89; MLR 0.92; RF 0.92; XGBoost 0.92	Ledger et al., 2023 (65)
Serum samples from the multimodal arm of the UKCTOCS trial	Borderline Cystic Pathology (BCP) and RNN	CA125, HE4, Glycodelin	AUC/Sensitivity: BCP (CA125 + HE4) 0.971/96.7%; BCP (CA125) 0.949/90.89%; RNN (CA125 + HE4) 0.987/96.7%; RNN (CA125) 0.953/92.1%.	Abrego et al., 2024 (79)
Retrospective multicentre clinical cohort	SVM; RF; kk-NN; Logistic Regression	CA125, CEA, CA199, CA72‐4, HE4	RF (AUC 0.86, Acc 99.82%); LR (AUC 0.95, Acc 78.0%); SVM (AUC 0.85, Acc 69.6%); KNN (AUC 0.90, Acc 74.8%)	Sun et al., 2024 (80)
Retrospective single-centre clinical cohort	CNN+U-Net	CA125, CA15‐3, CA72‐4, Estradiol, lipid levels (TG, LDL‐C/HDL‐C), CRP, Neutrophil‐to‐Lymphocyte Ratio,	AUC: 0.948, Sensitivity: 91.9%, Specificity: 86.9%	Feng et al., 2024 (81)
17 investigations; mixed clinical cohorts	RF; XGBoost; Neural Networks (including RNNs)	Tumor markers (CA125, HE4); inflammatory markers (CRP, NLR)	Diagnostic AUC >0.90 across studies; classification accuracy up to 99.82%; survival prediction AUC up to 0.866;	Hormaty S et al., 2025 (66)
Retrospective clinical cohort	RF, SVM, Decision Tree; Artificial Neural Network (ANN)	HE4, CA125, and NEU	Accuracy: RF >86%; SVM 85.25%; DT 82.91%; ANN 79.35% (10-fold CV).	Ayyoubzadeh SM et al., 2025 (67)
Retrospective clinical cohort	RF; SVM; GBM; Neural Network; Naive Bayes; Elastic Net; Logistic Regression; unsupervised clustering analysis	Peripheral blood biomarkers, including circulating tumor cells (CTCs)	RF showed superior performance (AUC 0.796 for advanced stage; AUC 0.809 for platinum sensitivity)	Xu, et al., 2025 (72)
Retrospective clinical cohort	Logistic regression	NLR, PLR, LMR, CA125; stratified by histologic subtypes of EOC	NLR (AUC 0.749), PLR (AUC 0.730), and LMR (AUC 0.709) showed significant predictive ability for EOC (all P < 0.001);	Li et al., 2021 (73)
Liquid biopsy samples with independent validation cohort	Transformer-based DL model (MethylBERT)	cfDNA methylation markers derived from genome-wide CpG sites	80% sensitivity and 95% specificity for early-stage EOC detection using the MethylBERT-based model; ddPCR assay retained good diagnostic performance	Li et al., 2024 (75)
Retrospective single-centre clinical cohort	CNN-based DL model (ResNet-50 backbone)	Ultrasound images; menopausal status; serum indicators	93.8% accuracy and AUC 0.983	Wang et al., 2024 (76)
Retrospective cohort across 3 hospitals	20 ML classifiers	51 laboratory tests and age; tumor markers (e.g., CA125),	AUC 0.949 in internal validation and AUC ≈0.88 in two external validation cohorts	Cai et al., 2024 (77)

### 3.2 EHR and multivariate models

Beyond imaging and molecular biomarkers, EHRs provide rich longitudinal real-world clinical data that can be leveraged by multivariate AI models for ovarian cancer prediction. EHRs capture patient demographics, laboratory results, comorbidities, treatment history, and healthcare utilization, enabling data-driven models to characterize disease risk and clinical outcomes in a more holistic manner (82). In ovarian cancer, EHR-based AI approaches can further incorporate prior treatment strategies, treatment-free intervals, performance status, and symptom information documented in clinical notes, thereby supporting individualized risk stratification and outcome prediction across the disease. Building on these advantages, EHR-driven AI models have been explored in several clinically relevant situations (83).

#### 3.2.1 Patient-level risk prediction and dynamic modelling using EHR data

EHRs are being utilized more and more to develop predictive models that find people who are at a higher risk of ovarian cancer by combining a lot of clinical data that is collected on a regular basis. Unlike traditional models that focus on single markers, EHR-based ML frameworks can accommodate demographics, comorbidities, symptoms, laboratory results, and other longitudinal health information, potentially enhancing early risk stratification (84, 85).

A recent study utilized statistical analysis and various ML models on routine clinical data from 349 individuals to facilitate early identification of ovarian cancer. The method used traditional statistical screening along with classifiers like RF, SVM, and gradient boosting models to find important serum tumor markers (like CA-125, CA19-9, Carcinoembryonic Antigen (CEA), and HE4) as well as inflammatory and biochemical blood indices. It was able to correctly tell the difference between malignant and benign ovarian tumors 91% of the time. These findings highlight the prospective efficacy of blood-based markers as supplementary tools for the early detection of ovarian cancer (86). Combining molecular tumor indicators with standard clinical and laboratory data is important because it makes the model much stronger and allows for a more full, multi-dimensional way to figure out how likely someone is to get ovarian cancer (87).

A DL-based survival model to forecast the risk of Venous Thromboembolism (VTE) in patients with EOC utilizing longitudinal EHR data. The model attained significantly superior predictive performance (AUROC 0.95-0.98) compared to traditional classification methods by integrating dynamic clinical information and considering competing risks. The system facilitated time-dependent, personalized risk assessment, demonstrating how EHR-driven DL models can aid in tailored complication risk forecasting and guide risk-based clinical treatments in ovarian cancer (84). Recent studies have shown how ML may use publicly available structured clinical datasets to combine demographic characteristics, tumor markers, and imaging-related aspects to predict ovarian cancer. For instance, a structured clinical dataset of 349 individuals was utilized to create a predictive model that distinguishes benign ovarian tumors from ovarian cancer using routinely gathered demographic and laboratory characteristics. Feature selection indicated HE4 and carcinoembryonic antigen (CEA) as the most informative indicators, with CEA offering supplementary value in patients with low HE4 levels. The resulting two-marker model exhibited enhanced discriminatory performance relative to the risk of ROMA, illustrating that succinct multivariable models can attain precise ovarian cancer risk categorization (78).

AI has been used to acquire patient-centered information that is vital for making clinical choices but is usually missing from structured datasets. It can also forecast risks and model outcomes. In ovarian cancer, patients’ values, preferences, and goals of care (GOC), encompassing attitudes towards treatment intensity and end-of-life care, are often recorded in free-text clinical notes instead of standardized areas. To fill this gap, recent research has used natural language processing (NLP) methods to get GOC-related information from unstructured EHR data (88). These methods blend organized clinical data with unstructured clinical notes to better understand what patients want, what they value, and their treatment goals. Text from clinical records is mapped to standardized medical concepts, such as Unified Medical Language System (UMLS) concept unique IDs, to ensure consistent interpretation across different records. To improve accuracy and reduce false positives, the results are reviewed manually and refined through repeated adjustments (89).

Together, these studies show that information drawn from EHR can be used to better identify high-risk patient groups, and can help guide preventive care and the management of complications in routine clinical practice.

#### 3.2.2 Insights from population-scale EHR modelling

Population-scale EHR studies suggest that longitudinal clinical history can capture weak pre-diagnostic signals at scale, supporting broader and potentially earlier risk stratification. AI models trained on longitudinal EHR data can identify how different clinical factors change and interact over time in ways that simpler risk models often fail to capture (90).

Conventional screening criteria rely on limited factors (e.g., age or selected risk indicators) and therefore miss many cases. Longitudinal EHR models aim to detect subtle pre-diagnostic patterns that are not captured by traditional risk variables. In the All of Us Research Program (>865, 000 participants), EHR-based models identified 3–6 times more true cancer cases across multiple cancer types, including ovarian cancer, than conventional indicators such as family history or known genetic variants (91).

Furthermore, neural network models trained on frequently gathered personal health data were created to categorize ovarian cancer risk in high-risk groups. The model exhibited moderate discriminative performance (AUC = 0.71) utilizing extensive population datasets and successfully categorized individuals into high- and low-risk groups, illustrating the potential of non-invasive, EHR-derived AI methodologies to enhance targeted ovarian cancer screening strategies (92). Large-scale EHR-based prediction models have shown that they can help find people who have a high chance of getting more than one type of cancer, like ovarian cancer. In order to do better than age and family background, these models use real-world data that is collected over time. These results show a lot more risk and help make it easier to use EHRs to predict risk in personalized, scalable cancer screening methods (93).

More recently, lightweight approaches that use only longitudinal medical history events have been explored for population-scale deployment. Can-SAVE, evaluated across 2.5 million adults from five regions, showed consistent gains in both retrospective (1.9M patients; 4-10× higher detection; AP 0.228 vs. 0.193) and prospective (426K patients; +91% detection; +36% coverage) studies, highlighting its effectiveness at scale. This study shows that using EHR data can support broader, cost-effective cancer detection across large populations, even within the practical constraints of real-world healthcare systems (94). This process goes beyond simple pattern recognition, capturing the intricate organization and temporal structure of patient health trajectories that may be missed by traditional methods.

#### 3.2.3 Incorporating patient-reported outcomes in AI models: role of data dictionaries and patient involvement

AI has been increasingly applied to capture patient values and preferences from diverse data sources, offering potential to enhance patient-centered care, while also facing challenges such as data quality, real-world validation, and ethical concerns (95). To ensure that AI models used in clinical settings accurately reflect patient experiences, it is essential to incorporate well-defined data dictionaries for PROs (96). A data dictionary provides a standardized framework for defining and coding variables, ensuring that patient-reported data is consistently interpreted across studies and clinical settings. This standardized approach is applied to other key outcomes, such as pain, fatigue, and emotional distress.

Pain score: A measure of patient-reported pain intensity, categorized as an ordinal variable using a 0–10 numeric scale, based on the PRO-CTCAE (97). Nausea: A treatment-related symptom, classified as either binary (Yes/No) or ordinal, with severity graded using a numeric scale, according to PRO-CTCAE (98). Fatigue/Emotional distress: A measure of the severity of fatigue/depression, recorded on a 1–5 Likert scale, as part of the PROMIS system (99). QoL-physical: Represents physical functioning status, quantified as a continuous variable using a normalized T-score, also part of PROMIS (100).

With the increasing use of EHRs, clinical information extraction (IE) plays a crucial role in automating data extraction and supporting clinical research (101). To facilitate this process, advanced systems like the Unified Medical Language System (UMLS) have been developed, which integrate over 2 million names for around 900, 000 concepts from more than 60 biomedical vocabularies and links concepts to external resources like GenBank (102).

Patient involvement in research has been shown to enhance the relevance and quality of healthcare studies. The Guidance for Reporting Involvement of Patients and the Public 2 (GRIPP2) checklist was developed to improve the transparency and consistency of reporting patient and public involvement in research. It includes both long-form and short-form versions, with 34 and 5 key items, respectively, covering aims, methods, outcomes, and the impact of involvement (103). Involving patients and stakeholders in research proposal reviews, as seen in the Patient-Centered Outcomes Research Institute (PCORI), showed that while initial scores varied from scientists, in-person discussions improved agreement among reviewers (104). The potential of AI is in enhancing personalized diagnostics for periodontal diseases, which often require early detection and individualized treatment approaches. Unlike traditional methods that use a “one size fits all” approach, AI can analyze diverse data sets, such as clinical records, imaging, and molecular information, to tailor treatments to each patient’s unique condition (105).

Data dictionaries not only ensure consistent data capture but also enable cross-study comparisons and data aggregation from diverse sources. Collaboration with patients and individuals with lived experience in developing these criteria ensures that the scales reflect real-world experiences, making the measures more relevant and accurate for clinical applications, and bridging the gap between clinical data and lived experiences in AI models.

## 4 Multimodal data fusion strategies

Multimodal AI represents a transition from precise prediction to clinically relevant decision support. Integrating diverse data types such as medical imaging, pathology results, molecular profiles, and clinical data helps achieve a more comprehensive understanding of ovarian cancer (106). Combining modalities often results in stronger predictors than relying on a single data source alone, and therefore, the development of effective multimodal AI models depends on how and when data is integrated (107).

### 4.1 Temporal multimodal integration: longitudinal data and disease dynamics

Data fusion in multimodal AI can occur at different stages, which are generally categorized as early fusion, intermediate fusion, and late fusion. These terms refer to when different data streams are combined in the model architecture (108).

#### 4.1.1 Late fusion: decision-level integration

In late fusion (sometimes called decision-level fusion), each modality is processed independently (often by its own specialized model) and the outputs or high-level features from those models are then combined (fused) to make a final prediction. Late fusion models have shown performance gains in ovarian cancer compared to uni-modal models (109).

For example, Zhao et al. integrated macroscopic imaging features derived from MRI radiomics, microscopic pathology features extracted from biopsy WSIs (pathomics), and immunohistochemical biomarkers (biopsy-adapted immunoscore) into a Cox-based nomogram to predict distant metastasis in locally advanced rectal cancer. The resulting multimodal model achieved strong discriminative performance (Concordance Index (C-index) ≈0.85 in the test cohort; 5-year AUC up to 0.95 in the training cohort) and consistently outperformed models based on any single modality, illustrating the effectiveness of decision-level integration of heterogeneous clinical data (110). Similarly, the radiologic and pathologic feature layers function as omics-like data modalities. Their AI-driven integration with clinicogenomic predictors at the decision level mirrors multi-omics fusion strategies increasingly used for prognostic modeling, underscoring the role of multimodal AI systems in precision oncology decision making of HGSOC (111). Recent work introduced a foundation model-driven multimodal framework (FoMu) that integrates clinical variables, MRI data, and H&E whole-slide pathology images for prognostic prediction in HGSOC. The FoMu method used radiologic and pathologic foundation models that had already been trained to extract features and mix modality-specific and cross-modal representations. This led to accurate predictions of overall and progression-free survival across multiple center cohorts, demonstrating how AI-driven multimodal integration could enhance individual risk classification and improve therapeutic decision-making in ovarian cancer (112).

#### 4.1.2 Intermediate fusion: feature-level modeling

Intermediate fusion strikes a compromise: each modality is initially processed on its own (like late fusion) to extract intermediate features, but then those features are fused in a joint model layer that continues learning. For example, one might use a CNN to convert a pathology image into a 128-dimensional feature vector, use a separate model to convert genomic data into another feature vector, and then concatenate those vectors and feed them into a fully-connected neural network or transformer that produces the final prediction. The fusion happens in the middle of the pipeline, hence “intermediate.” This approach allows some pre-training or dimensionality reduction per modality (which is helpful if the dataset is small) while still allowing the model to learn interactions between modalities at the feature level (113).

Many advanced multimodal architectures fall into this category. For example, most image-based prognostic models use either histology or radiology alone, in part due to the gigapixel scale of WSIs and the large spatial mismatch between pathology and radiologic images. Hierarchical Multimodal Co-Attention Transformer (HMCAT) addresses these challenges through a weakly supervised, interpretable multimodal transformer that hierarchically encodes histologic features and integrates them with radiologic representations via co-attention, enabling feature-level fusion and improved survival prediction across multiple cancer datasets, in which modality-specific representations from histology and radiology are fused at the feature level via co-attention before final prognostic prediction (114). Autoencoder-based approaches are often categorized as intermediate fusion methods, as heterogeneous omics layers are mapped into a shared latent representation that serves as an intermediate feature space for downstream analysis. Using this strategy, Variational Autoencoder (VAE) and Maximum Mean Discrepancy Variational Autoencoder (MMD-VAE) based frameworks have been applied to ovarian cancer, where integrated mono-, di-, and tri-omics data were embedded into a common latent space for molecular subtype classification and survival analysis, achieving high subtype classification accuracy (up to ~95%) and robust prognostic stratification (115).

#### 4.1.3 Early fusion: input-level joint representation

In early fusion, data from different modalities are combined at the input or very early in the model, creating a single joint feature space that the model learns over. For example, one could concatenate a patient’s genomic feature vector with her radiomic features before feeding them into a neural network, or even attach image pixels and genomic data as parallel channels into a unified model.

In practice, however, early fusion requires substantially more training data to tune the large number of parameters such models entail. With many modalities, the dimensionality of the joint input explodes, and the model must learn to calibrate disparate data types (image intensities vs. gene counts, for instance) which can be challenging. As a result, early fusion remains largely exploratory in ovarian cancer due to limited availability of fully matched multimodal cohorts (116).

HEALNet represents an early-fusion multimodal architecture, in which heterogeneous raw inputs from whole-slide histopathology and multi-omic data are jointly integrated at the input level to learn a shared latent representation for survival analysis (117). Another study proposed an early-fusion DL framework based on a denoising autoencoder, in which multi-omics data from TCGA were jointly embedded at the input level into a shared low-dimensional latent space. These fused representations were subsequently used for k-means clustering to define molecular subtypes, followed by construction of a lightweight L1-penalized logistic regression classifier. Using this approach, the authors identified 34 subtype-associated biomarkers and 19 enriched Kyoto Encyclopedia of Genes and Genomes (KEGG) pathways. Robustness was demonstrated through independent validation across three Gene Expression Omnibus (GEO) datasets, where the derived subtypes showed consistent predictive performance. Literature curation further confirmed that 19 of the 34 biomarkers (56%) and 8 of the 19 KEGG pathways (42.1%) had previously been implicated in ovarian cancer, supporting the biological relevance of the early-fusion multi-omics subtyping strategy (118). Another early-fusion CNN-Transformer model achieved higher performance than single CNN or Vision Transformer (ViT) models for multiclass ovarian tumor classification on transvaginal ultrasound, with an AUC of 0.990, accuracy of 92.1%, sensitivity of 92.4%, and specificity of 98.9%, supporting its suitability for clinical decision support (119).

**Figure 4:**
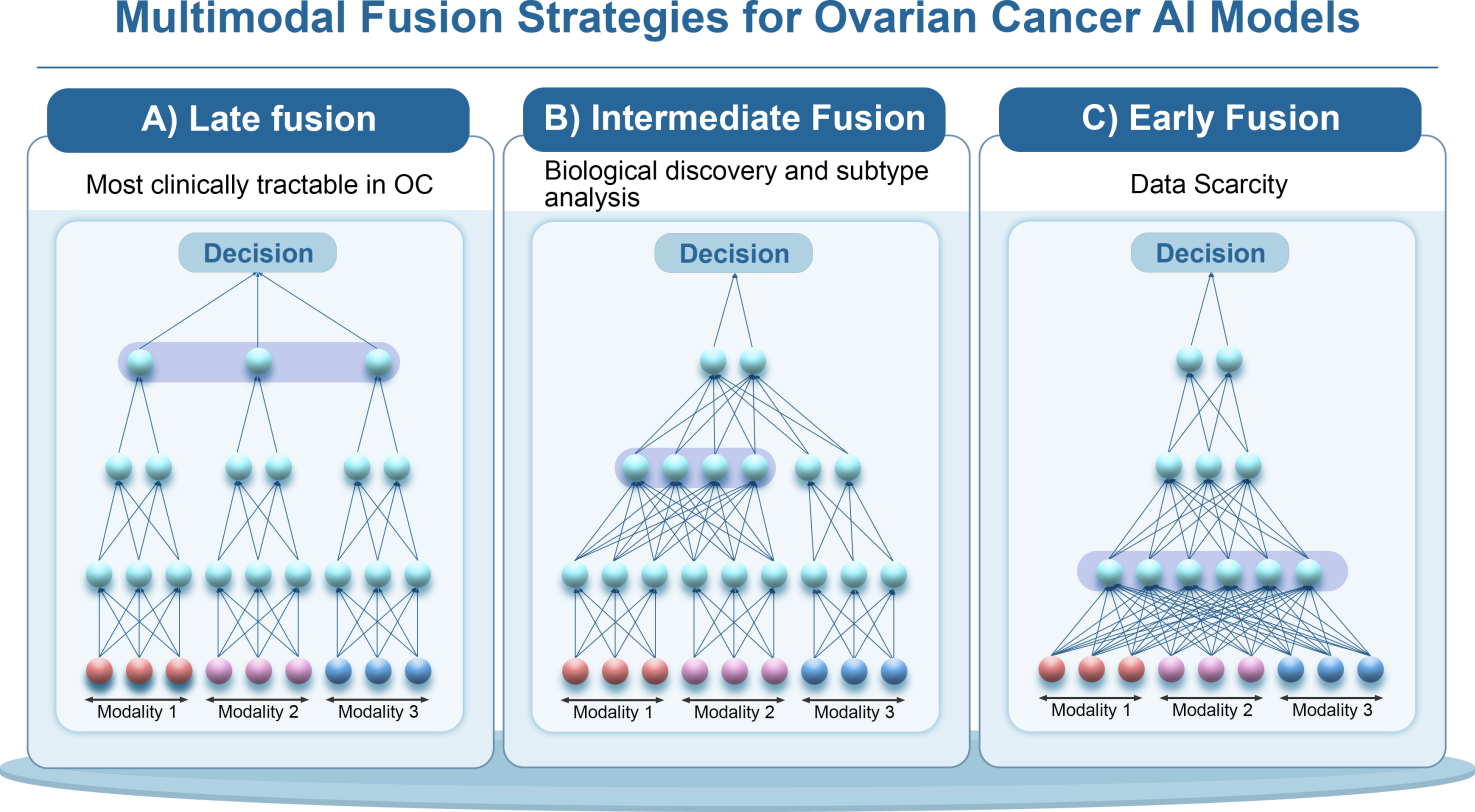
Temporal multimodal integration strategies for ovarian cancer AI models. Temporal multimodal integration strategies for AI models in ovarian cancer, including: **(A)** Late Fusion: Processes each modality independently with separate models, and combines their outputs at the decision level to produce the final prediction. This strategy allows independent optimization of each modality-specific model before aggregation. **(B)** Intermediate Fusion: Each modality is processed independently to extract intermediate features, which are then combined at a later stage. This approach enables feature-level fusion, allowing the model to learn interactions between modalities after pre-processing. **(C)** Early Fusion: Combines data from different modalities (e.g., clinical, imaging, genomic) at the input stage, creating a joint feature space that the model learns from. This approach requires large datasets due to the high dimensionality of the combined inputs.These strategies enhance the model’s ability to handle multimodal data and improve diagnostic, prognostic, and therapeutic decision-making in ovarian cancer.

To sum up, multimodal fusion is a key part of AI in ovarian cancer as we progress toward precision treatment. AI models can use the complementary information from radiologic, pathologic, genomic, and clinical data to capture both the “macro” tumor traits that can be seen on scans and the “micro” molecular factors that dictate behavior. Thoughtful choice of fusion strategy (early vs. late) and model design can mitigate data limitations and highlight informative cross-modal patterns. To provide a systematic methodological overview, we summarize the major paradigms of multimodal fusion strategies used in AI-driven ovarian cancer research (**Table 3**). From a translational perspective, early fusion remains largely exploratory due to data scarcity, intermediate fusion is most suitable for biological discovery and subtype analysis, whereas late fusion currently represents the most clinically deployable paradigm in ovarian cancer (**Figure 4**).

**Table 3:** Overview of temporal integration and representative implementations.

Fusion strategy	Classification criterion	Method category	Typical implementations	Represent example
Early fusion	Model architecture	Direct modeling	Fully Connected (FC) Layer/CNN/RNN	Hemker K et al., 2024 (117)
		Autoencoder-based modeling	Regular/denoising/stacked/VAE	Guo et al., 2020 (118)
Intermediate fusion	Unimodal branch design	Homogeneous branches	Marginal/joint	Hira MT et al., 2021 (115)
		Heterogeneous branches	Marginal/joint	Li et al., 2023 (114)
Late fusion	Output aggregation scheme	Averaging-based fusion	Equal/weighted	Deng et al., 2020 (120)
		Meta-learning-based fusion	Weighted	Zhao et al., 2025 (110)

### 4.2 Spatial omics and AI: mapping tumor-immune ecosystems

**Figure 5:**
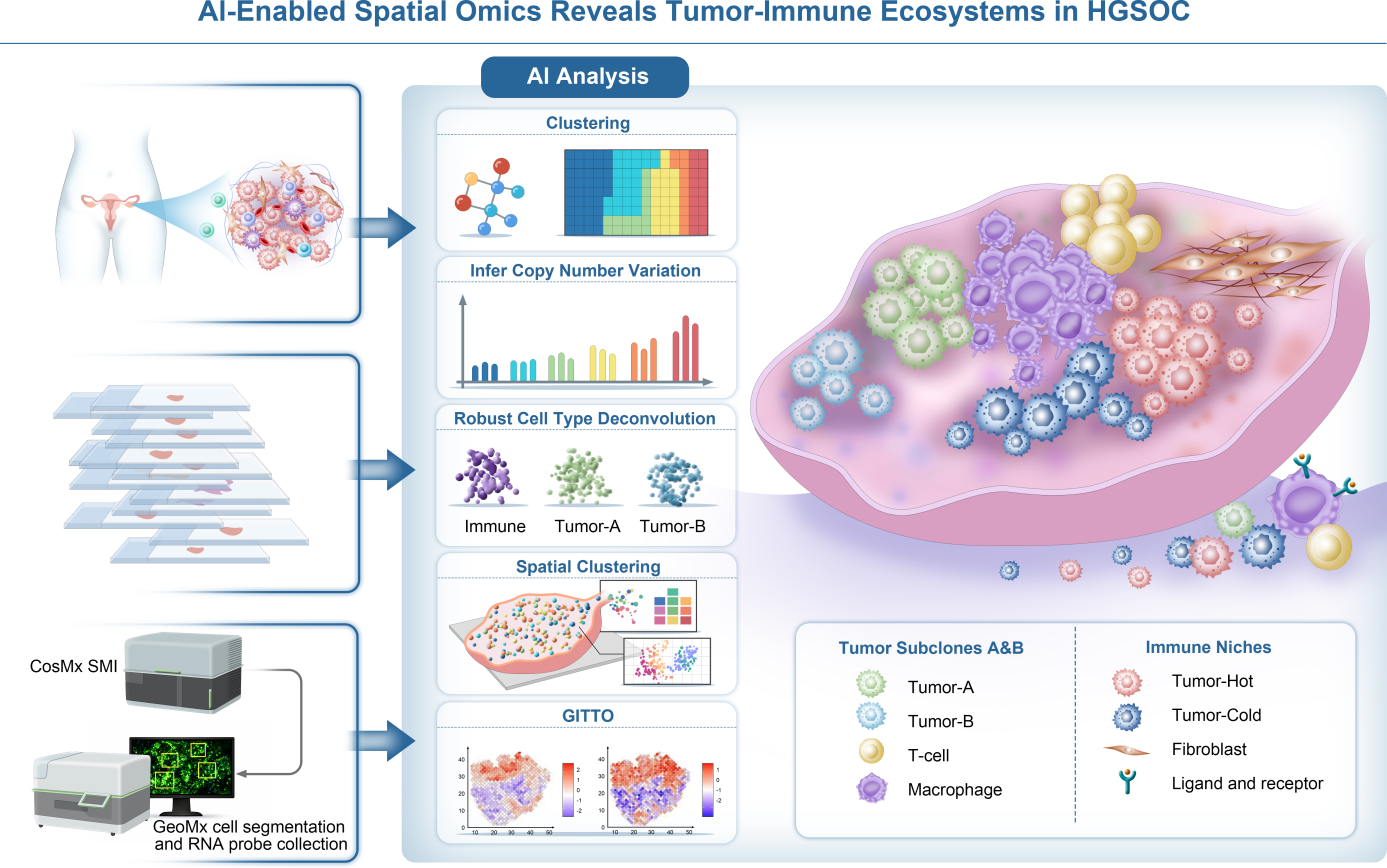
AI-enabled spatial analysis reveals tumor-immune ecosystems. Spatial omics data generated from multiplexed imaging and transcriptomic platforms (e.g., CosMx SMI and GeoMx) are analyzed using AI-assisted computer method. These analyses include unsupervised clustering, inference of copy number variation, robust cell-type deconvolution, and spatial clustering to delineate tumor subclones and immune cell populations. Integration of spatially resolved gene expression with tissue architecture enables the identification of distinct tumor subclones (Tumor-A and Tumor-B), immune niches (tumor-hot and tumor-cold regions), and ligand-receptor interactions within the tumor microenvironment, providing a comprehensive view of tumor-immune ecosystem organization.1. Data Acquisition: High-resolution tissue images and spatial transcriptomics data are captured to map gene expression across tissue sections. 2. Spatial Mapping: AI models map the locations of tumor and immune cells (e.g., T-cells, macrophages) within the tissue, identifying areas of immune activation or suppression. 3. Cell Type Deconvolution: AI models like Tangram and Cell2location deconvolve the data to estimate the abundance and distribution of different cell types across the tissue. 4. Gene Expression and Tumor Microenvironment Mapping: Gene expression data are integrated with tissue morphology, revealing insights into tumor progression, immune evasion, and the tumor microenvironment. 5. Tumor-Immune Interaction Mapping: AI analysis uncovers interactions between tumor and immune cells, providing insights into immune activation, suppression, and potential therapeutic targets.

Spatial omics extends multimodal AI from integrating “different data types” to integrating “different spatial contexts”, enabling AI to model not only what genes are expressed, but where and with whom. While bulk genomics provides important averages of tumor molecular traits, spatial omics technologies add a new dimension by preserving the spatial context of gene/protein expression within tissue sections (121). In ovarian cancer, AI-spatially resolved data are illuminating how cancer cells and immune cells are organized in the tumor microenvironment (**Figure 5**).

AI plays a crucial role in analyzing complex spatial datasets and integrating them to map tumor-immune ecosystems and subclonal structures with greater detail. Developing AI frameworks that are both interpretable and capable of integrating data-driven and mechanistic approaches will be key to unlocking the biological insights and clinical potential of large-scale spatial tissue data (122).

#### 4.2.1 Spatial outputs and multimodal integration in AI models

Spatial omics data can be integrated with other modalities, such as single-cell multi-omics, proteomics, imaging, and clinical features, through various strategies to construct complex AI/ML models.

Pair mapping and deconvolution: By using single-cell RNA-seq data as a reference, it is mapped to spatial data. Representative methods include Tangram (DL) for aligning scRNA and spatial transcriptomics) (123), Cell2location (Bayesian model integrating scRNA cell types with spatial expression) (124), and RCTD. Neural networks or Bayesian frameworks are then used to map (deconvolve) single-cell types or expression distributions to spatial locations, outputting cell type abundance or single-cell level expression at each spatial point.

Graph Neural Network (GNN) fusion: SpatialGlue is an AI-driven GNN model that integraes spatial omics data with a dual-attention mechanism to accurately decode spatial domains and cellular properties across multiple omics modalities.

Imaging-omics integrated learning: Using high-resolution tissue images to extract morphological features, which are then combined with spatial transcriptomics data as inputs to a model. For example, DL model StLearn/SpaCell utilizes convolutional networks to extract spatial features from tissue section images and performs smoothing or clustering alongside gene expression data (125, 126).

The outputs of spatial biology include cell-cell interactions, spatial gene expression, and tumor microenvironment maps, which help reveal molecular heterogeneity, cell interaction networks, and functional states within tissues and tumors (127), further enhancing our understanding of complex biological systems.

#### 4.2.2 Spatial transcriptomics for subclone mapping

HGSOC are known to be spatially heterogeneous, often containing multiple distinct clones of tumor cells intermixed with stroma and immune infiltrates. In 2024, a work has demonstrated that AI-guided spatial transcriptomic analysis can help interpret prognostic regions detected by DL in HGSOC. By integrating image-based AI models trained on H&E slides with spatially resolved transcriptomics, Laury et al. showed that AI-identified tumor regions, exhibit distinct transcriptional programs compared with background tumor areas, provides the proof of concept that AI-informed spatial transcriptomics can uncover biologically and clinically relevant intratumoral heterogeneity beyond standard histopathologic assessment (128).

Then A landmark study by Denisenko et al. in 2024 applied spatial transcriptomics to HGSOC and revealed extensive intratumoural heterogeneity driven by genetically distinct tumor subclones in ovarian cancer. Using 10x Genomics Visium, this work demonstrated that multiple subclones with different copy number alteration profiles coexist within individual tumor sections and occupy distinct spatial niches. These subclones exhibited differential ligand-receptor expression patterns and preferential associations with specific stromal and immune cell populations. High-resolution validation with CosMx single-molecule imaging further confirmed subclone-specific interactions with immune cells, fibroblasts, and endothelial cells, as well as the presence of distinct autocrine signaling loops. Using an AI-enabled computational framework integrating unsupervised expression clustering, copy number variation inference (inferCNV), probabilistic cell-type deconvolution (RCTD), graph-based spatial neighborhood analysis (Squidpy), and ligand-receptor network inference (connectomeDB/NATMI), AI clustering of spatial gene expression was able to delineate these micro-domains, and suggest why certain clones might resist therapy (e.g. a clone shielded by immunosuppressive stroma) (129).

Moreover, spatially resolved transcriptomic and proteomic analyses have revealed that the progression from serous borderline tumors to low-grade serous carcinoma occurs through an epithelial micropapillary intermediate state and is supported by coordinated tumor-stroma interactions. Integrated spatial profiling identified actionable targets, with combined Cyclin-Dependent Kinase 4/6 (CDK4/6) and FOLR1 inhibition demonstrating therapeutic efficacy *in vivo* (130).

This advanced AI approach captures both the spatial organization and the molecular dynamics within the tumor, which are critical for improving therapeutic strategies and predicting treatment resistance. Together, these findings highlight the value of AI-enabled spatial multi-omics for understanding therapy resistance and identifying actionable therapeutic targets.

#### 4.2.3 Spatial proteomics and microenvironment

Complementary to spatial RNA, spatial proteomics (e.g. imaging mass cytometry, Cytometry by Time-of-Flight (CyTOF), multiplex immunofluorescence) can map dozens of proteins at single-cell resolution on tissue sections (131, 132).

A 2025 study by Makhmut et al. applied deep spatial proteomics to Serous Tubal Intraepithelial Carcinomas (STICs) and matched invasive HGSOCs, quantifying over 10, 000 proteins *in situ*. The analysis revealed striking proteomic similarity between STICs and invasive tumors, indicating that many malignant programs are established at the precursor stage. Despite this similarity, two distinct spatial subtypes were identified, characterized by divergent tumor-immune microenvironments, including immune-enriched versus fibrotic, matrix-remodeling niches, and differences in cell-of-origin markers. AI-based clustering of spatial neighborhoods further uncovered early microenvironmental remodeling and highlighted subtype-specific vulnerabilities, such as aberrant activation of cholesterol biosynthesis pathways. Functional validation showed that inhibition of DHCR7 suppressed ovarian cancer cell growth and synergized with carboplatin, illustrating how AI-enabled spatial proteomics can uncover targetable pathways emerging from early tumor-stroma interactions (133).

Another spatial proteomic study (Yeh et al., 2024) established a large-scale single-cell spatial transcriptomic atlas of high-grade serous tubo-ovarian cancer by profiling ~2.6 million cells across 130 tumors, enabling quantitative mapping of tumor-immune spatial organization. Using AI-enabled computational analysis, including DL-based cell segmentation (Mesmer), unsupervised embedding/clustering (UMAP) to derive immune infiltration-associated cell states, and machine-learning classifiers as SVM to predict T/NK infiltration from malignant-cell programs, the authors identified a malignant transcriptional program that robustly marks the presence of TILs (MTIL) that robustly tracks spatial immune infiltration and is associated with clinical outcome. Integrating spatial cell-state mapping with perturbational transcriptomics (Perturb-seq), they further pinpointed genetic regulators (e.g., PTPN1 and ACTR8) that drive immune-evasive malignant states; genetic knockout or pharmacologic inhibition (PTPN1/PTPN2 inhibitor) increased susceptibility of ovarian cancer cells to T and NK cell cytotoxicity. Collectively, this work illustrates how AI-assisted spatial transcriptomics can connect spatially patterned tumor cell states to immune microenvironment exclusion and nominate actionable regulators of immune evasion (134).

In summary, integrating spatial omics with AI provides valuable insights into ovarian cancer heterogeneity, highlighting how cancer and immune cells interact and coexist. These approaches are uncovering new substructures (subclones, niches) in ovarian tumors that were invisible to bulk analyses, and they offer a path toward *spatially-informed precision therapy*, treating not just an “average” tumor, but the specific malignant ecosystems present in each patient.

Spatial AI marks a conceptual shift: from predicting outcomes to explaining why tumors behave differently within the same patient in ovarian cancer. **Table 4** summarizes representative multimodal AI data fusion strategies that have been applied to ovarian cancer.

**Table 4:** Representative AI-based multimodal data fusion strategies in ovarian cancer.

Modality combination	Common models	Model type	Performance	Reference
Ultrasound imagine + clinical data	UMORSS	CNN + attention-enhanced convolution+XGBoost	0 FN; AUC 0.955/0.926 (int./ext.); reader study: AUC + 10.58%, sensitivity +22.48%.	Wang X. et al., 2025 (7)
Ultrasound image + clinical data	OTC-NET	CNN ensemble (DenseNet201/ResNet34/MobileNetV2), RF (2-stage, decision-level)	AUC 0.81 (95% CI 0.72-0.89); Acc 0.733; Sen 0.660; Spe 0.800. AI-assisted radiologists: AUC + 0.09 (junior, to 0.71) and +0.08 (senior, to 0.76).	Liu P. et al., 2025 (135)
MRI radiomics + clinical data	Radiomics-clinical model	Supervised ML	Radiomics AUC 0.65; Combined AUC 0.77 (mean 0.7667 ± 0.0217), Acc 0.7079 ± 0.0647, Sen 0.5429 ± 0.1432, Spe 0.7936 ± 0.1047 (5-fold CV)	Na I. et al., 2024 (136)
MRI radiomics + pathology + clinical data	MHA_CHP multimodal model	MRI habitat radiomics (K-means habitat + PyRadiomics RF habitat model) + Pathology (ViT patch-level + MIL (PLH/BoW) RF WSI-level model) + Clinic (logistic regression) Multi-Head Attention (Transformer-based fusion); compared with stacking ensemble (LR meta-learner)	MHA_CHP AUC 0.789 (internal validation), 0.807 (external test)	Bi Q. et al., 2025 (137)
CT radiomics+ clinical data	Preoperative debulking prediction model/PIV scoring system	univariate + multivariable logistic regression (stepwise forward selection)	AUC 0.788 (95% CI 0.720-0.856) accuracy 85.47%, specificity 100%	Gu Y. et al., 2021 (138)
CT radiomics+ clinical data	CT-based cytoreduction prediction model	univariate + multivariable logistic regression	Best model (DPT + large-volume ascites): PPV 68%, Sens 52%, Spec 90%; DPT alone: PPV 57%, NPV 85%; inter-reader agreement for DPT 93%	Dowdy S.C. et al., 2004 (139)
H&E WSI + CT radiomics + genomics (HRD/HRP) + clinical data	multimodal Cox framework (RH/GRH/GRHC)	H&E: ResNet-18 nuclei/tissue features: Cox; CT: Cox; Genomics: HRD/HRP rule label; Clinical: Cox	Test c-index: RH (CT+H&E) 0.62; GRH (Genomics+CT+H&E) 0.61; unimodal: H&E 0.54, CT 0.53, HRD 0.52, clinical 0.51. GRH risk groups significant for OS (P = 0.023) and PFS (P = 0.040) in test set	Boehm KM et al., 2022 (111)
H&E WSI + molecular biomarkers (BRCA/HRD)	PathoRiCH (DS-MIL)	Weakly supervised DL: Dual-stream MIL with self-supervised ResNet-18 (SimCLR) feature extractor, attention-based MIL aggregator; integration with BRCA/HRD via Cox/KM analysis	AUC-ROC 0.596 (internal SEV), 0.602 (TCGA), 0.593 (SMC) TCGA HR 1.95, 95% CI 1.35-2.81	Ahn B. et al., 2024 (140)
CT radiomics+ clinical data	Radiomics-based ML classifier	SVM with Gaussian RBF kernel	AUC 0.806 ± 0.078; Acc 83.3%, PPV 81.8%, NPV 83.9%	Zhang K. et al, 2024 (141)
MRI radiomics + clinical data + H&E WSI	FoMu	Pretrained encoders + attention	Clinic+MRI+WSI: C-index OS 0.845 (internal), 0.836 (external A); PFS 0.877 (internal), 0.848 (external A).	Bi Q. et al., 2025 (112)
H&E WSI + genomics	MCAT	Transformer-based co-attention, Cox	c-index 0.653, outperforming unimodal WSI and genomics baselines by 3.0-6.87%	Chen RJ. et al., 2021 (142)

Beyond ovarian cancer-specific studies, multimodal AI frameworks developed in other cancer types transferable to ovarian cancer research (**Table 5**). These frameworks illustrate what is technically possible for ovarian cancer.

**Table 5:** AI-based multimodal strategies with potential translational value for ovarian cancer.

Modality combination	Common models	Model type	Performance	OV Data Used?	Transferability to Ovarian Cancer	Reference
CT + pathology	SMuRF	Swintransformer	C-index = 0.81 (DFS); AUC = 0.75 (grade); independent DFS predictor (HR = 17, p < 0.0001)	No	Relatively modest	Song et al., 2025 (143)
Clinical + Genomic	Surv_GCNN	GCN+Cox	Best performance in 7/13 TCGA cancers; BRCA median C-index = 0.78.	Yes	Medium	Ricardo. et al., 2021 (144)
Clinical + Multigenomic + WSI	MultiSurv	CNN + FC networks (MLP)	33 TCGA cancer types, with highest performance for clinical + mRNA fusion (Ctd = 0.822).	Yes	High	Vale-Silva et al., 2021 (145)
Clinical +Multigenomic + PPI	DeepMOCCA	GCN + self-attention + Cox	Across 33 TCGA cancer types achieved strong C-index (e.g., OV = 0.85, LUAD = 0.88, BRCA = 0.86)	Yes	High	Althubaiti S et al., bioRxiv 2021 (146)
Multigenomic (mRNA + DNA methylation + miRNA)	MOGONET	GCN+VCDN	ROSMAP AUC = 0.874、LGG AUC = 0.840、BRCA ACC = 0.829(mean ± SD over 5 splits	No	Relatively modest	Wang et al., 2021 (147).
H&E + Multigenomic	Multimodal CustOmics	GNN+VAE+Hierarchical Mixture-of-Experts+Cox	Across 33 TCGA cancer types, consistently outperforming WSI-only and omics-only baselines (e.g., pan-cancer survival C-index up to ~0.84).	No	Medium	Benkirane H. et al., 2025 (148)
RNA-seq + Multigenomic+ GDC/TCGA+TARGET	OmiEmbed	VAE+GradNorm+FC networks (MLP)	Survival prediction on GDC achieved C-index = 0.7715 and IBS = 0.1657 (best among 10 baselines); multi-task training further improved to C-index = 0.7823 and IBS = 0.1590.	Yes	High	Zhang et al, 2021 (149)

“Modalities” lists the input data each model uses. “OV Data Used?” indicates if ovarian cancer cases were included in training. The Transfer Rating (High/Med/Low) reflects how well the model is expected to work for OV, and the justification cites our sources and domain reasoning. Model type reflects the primary architectural or learning paradigm; many models are hybrid.

## 5 Model interpretability, generalizability, and clinical translation

As new tools are developed for ovarian cancer, questions have shifted toward how their results are obtained and whether they can be used safely in real-world care. However, making these tools understandable, reliable across different patient groups, and practical for routine use remains challenging (150).

### 5.1 Clinical trustworthiness and model reliability

#### 5.1.1 Interpretability and explainable AI

The application of AI models in ovarian cancer has rendered interpretability a crucial necessity for clinical reliability and secure implementation. Nonetheless, the lack of transparency particularly in medical settings, drives efforts to make their decision-making processes easier to understand and trust (151). Recent research had shown that interpretability alone may not be enough for clinical usage, even though explainable AI (XAI) provides technological ways to show and attribute model predictions. The concept of causal interpretability has been established to distinguish system-level explainability from the human capacity to understand, contextualize, and reason causally about model outputs. From this perspective, explainability refers to a trait of the AI system, whereas causal understanding signifies the caliber of explanations acknowledged and utilized by physicians. This distinction is especially relevant in medicine, as AI-generated explanations should improve clinical rather than merely disclose internal model attributes (152).

#### 5.1.2 Robustness and bias

Bias can arise at any stage of medical AI development, from data collection and labeling to model training, validation, and clinical use. If these biases aren’t dealt with, they could affect clinical judgments and make current healthcare disparities worse, especially in high-risk clinical situations. Bias can occur because to skewed or inadequate datasets, incorrect labeling, an undue emphasis on aggregate performance metrics, or reduced accuracy when models are applied to patient cohorts that differ from those utilized during development. To reduce these dangers, individuals need to use a range of representative data, look at how well each subgroup does, make model outputs clearer, report any apparent biases clearly, and do thorough testing before applying them in clinical settings (153).

Although XAI have improved model transparency, the reliability of current explanation methods remains a concern. Research indicates that little alterations in input data might result in significantly divergent explanations. Such inconsistency can make clinicians less sure of themselves and make it harder to make clinical decisions. These discoveries underscore the necessity to assess explanatory methodologies in the context of realistic data variability and to prioritize those that produce consistent outcomes prior to clinical application (154).

These findings underscore the necessity to limit bias and ensure clear, trustworthy and equitable use of AI in practice.

#### 5.1.3 Human-AI collaboration

The goal of current AI solutions for ovarian cancer is to help clinicians make better judgments, not to take their place. The multimodal, uncertainty-aware AI framework for assessing ovarian cancer risk demonstrates that human-AI collaboration yields superior outcomes compared to individual (155).

Recent developments in AI have prompted a shift from technology-centric approaches to human-centered AI (HCAI), which emphasizes the design of AI systems to better meet human needs. HCAI focuses on human control, responsibility, and empowerment when using AI-powered technology, rather than just how well the algorithms work. Researchers and practitioners may guarantee that AI systems augment human skills, promote well-being, and provide a positive impact on society by integrating human-centered principles in the design, development, and evaluation of AI. This way, AI systems don’t take over or get rid of human tasks (156). From a human-centered AI point of view, these kinds of systems can be thought of in terms of their goal (augmentation, automation, and autonomy), values (ethics, safety, and performance), and attributes (oversight, comprehensibility, and integrity). This approach supports doctors in making better decisions, but doctors remain responsible for the final call (157).

These approaches together make ovarian cancer AI look more like a system that gather doctors work together rather than a replacement for human.

### 5.2 Generalization and pathways to clinical deployment

#### 5.2.1 Generalizability and domain shift

Differences between research datasets and real-world clinical data sometimes lead to worse results, which makes it impossible to use these methods in real life (158).

Differences in how WSIs are collected, how tissue samples are stained, and where the data come from can lead to shifts in data characteristics, making it difficult for pathology analysis methods to perform reliably across different hospitals and datasets (159). The WSI-P2P architecture was presented to tackle this difficulty as a domain-robust whole-slide analysis method that reduces performance decline across diverse datasets. The consistent performance in both intra- and inter-domain evaluations underscores the promise of scalable multiple-instance learning frameworks to enhance robustness against domain shifts in practical histopathology applications (160). Limited reliability of medical imaging tools across clinical settings arises from both overfitting and insufficient constraints, underscoring the need for rigorous evaluation in real-world settings (161).

The transition of domains through strong modeling and rigorous evaluation is vital for reaching dependable and clinically useful AI.

#### 5.2.2 Federated learning and privacy

The acquisition of adequately vast and diversified datasets is frequently obstructed by privacy and data ownership limitations. FL has emerged as a solution by allowing AI models to be trained collaboratively on data from multiple institutions without the data ever leaving its source site (162). In FL, a common model is initiated and sent to each institution, where it is locally trained on that site’s data; only the model weight updates (and not the raw data) are then sent back and aggregated to form an improved global model while keeping patient data private (163). In ovarian cancer, FL is increasingly used to combine WSIs from multiple hospitals, addressing the limited availability of rare subtypes at individual sites. HFed-MIL is a heterogeneous federated multiple-instance learning framework for ovarian cancer pathology that enables cross-institutional model training under privacy constraints, improves robustness to site-specific heterogeneity, and maintains model interpretability (164).

Researchers are addressing these by developing adaptive federated optimizers and strategies like FedProx, FedAvg improvements, as well as ensuring each site’s contribution is weighted properly. Privacy is further safeguarded in FL by techniques such as differential privacy (adding noise to updates so individual patient contribution is obfuscated) and secure multi-party computation, as highlighted by the systematic review (165) These ensure that even the model updates don’t inadvertently leak patient info.

We foresee FL becoming standard in multi-institutional studies, and possibly a requirement for regulatory approval that a model has been trained/validated on diverse patient data.

#### 5.2.3 Toward clinical deployment

Despite rapid methodological advances, most AI models developed for ovarian and pancreatic cancer have not yet been translated into clinical practice, largely due to limited and non-public imaging datasets, low disease prevalence, and the absence of Food and Drug Administration (FDA)-qualified imaging biomarkers (166). These challenges are intensified by concerns about data quality, bias, and transparency, highlighting the need for more interpretable and reliable models for rare cancers.

Translating laboratory findings into clinically applicable tools requires rigorous clinical validation, clear definition of their capabilities and limitations, and adherence to regulatory standards (166, 167).

**Figure 6:**
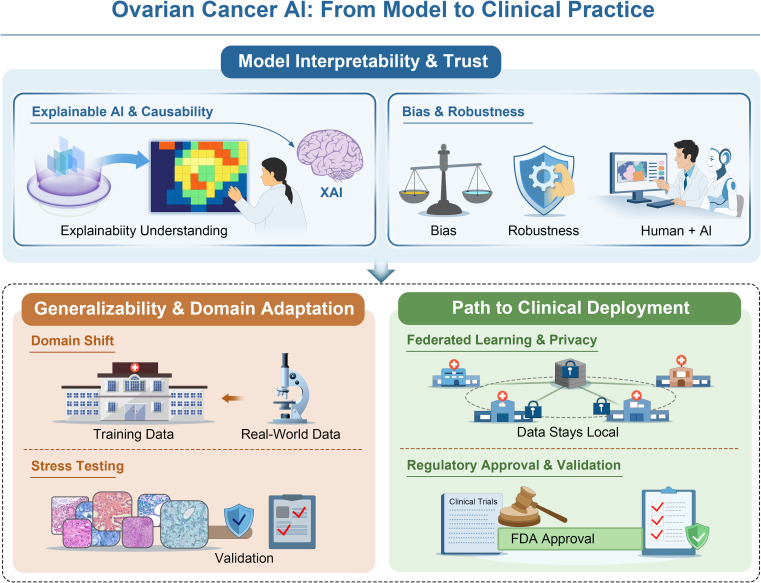
Model interpretability, generalizability, and clinical translation of AI. This schematic illustrates key components required to translate AI models for ovarian cancer into clinical practice. Model interpretability and trust are enhanced through explainable AI and causability analyses, together with systematic assessment of bias, robustness, and human-AI interaction. Generalizability and domain adaption is addressed by evaluating domain shift between training and real-world data, complemented by stress testing and multi-cohort validation. Path to clinical deployment is supported by privacy-preserving deployment strategies, such as FL, and by rigorous regulatory validation and approval processes. Collectively, these elements define a pathway toward reliable, safe, and clinically actionable AI applications.

In summary, translating a promising AI model into an approved clinical tool for ovarian cancer requires an emphasis on explainability (to foster clinician trust and guarantee patient safety), robustness (to manage real-world variability), collaborative design (to enhance clinician expertise rather than circumvent it), and stringent clinical validation (**Figure 6**).

## 6 Discussion

Although predictive performance in ovarian cancer research has improved substantially, accuracy alone is insufficient to ensure clinical relevance. Many approaches have limited practical impact without explicit linkage to clinical decision-making.

### 6.1 Clinical readiness and realistic scope of current applications

Currently, the AI applications most proximate to clinical implementation are those that conform to existing diagnostic procedures, especially image-based triage and digital pathology. Standardized inputs, clear clinical queries, and dependable reference standards are helpful for tasks like classifying adnexal masses and analyzing whole-slide histopathology. In these situations, AI is mostly used to help people make decisions. It makes things more consistent, efficient, and easier to get expert-level assessments, especially when resources are limited. These systems work best when they add to, rather than replace, the judgment of the clinician.

### 6.2 Clinical decision-making as the measure of value

A high level of prediction accuracy is not enough to make therapeutic use acceptable. For AI outputs to have clinical significance, they must facilitate practical decisions, such referral prioritizing, treatment planning, or follow-up tactics, and exhibit benefits on clinically pertinent outcomes. These include cutting down on unneeded operations, making risk classification better, or making it possible to intervene sooner. To show that anything is better than present standards of care, it needs to be tested in the future and in the actual world.

### 6.3 Exploratory approaches and translational barriers

Early-fusion multimodal models and pan-omics frameworks are still mostly in the testing stage. These methods are based on biology, but they have problems since the sample sizes are small, the modality overlap is not complete, and it is hard to combine the data. Currently, they are more appropriate for hypothesis formulation and biological understanding than for standard clinical application. The main problems with translation between modalities are not model complexity or performance, but external validation, interoperability, interpretability, and integration into clinical workflows.

### 6.4 Toward responsible clinical translation

Successful clinical deployment of AI in ovarian cancer will require more than technical advances. Ethical considerations, bias mitigation, data governance, and patient privacy must be addressed alongside regulatory and implementation challenges. Approaches such as FL, explainable AI, and human-centered design provide practical pathways to address these issues. Progress will be contingent upon the alignment of AI development with well-defined cases and the assessment of systems across varied patient demographics and healthcare environments.

Taken together, the greatest impact in ovarian cancer care is likely to arise from careful integration of AI into existing clinical workflows, where its strengths are leveraged and its limitations are clearly understood. To highlight key aspects of the current research, **Box 1** summarizes the criteria for evaluating clinical data in ovarian cancer AI models, **Box 2** outlines the limitations of integrating diverse datasets, and **Box 3** presents potential future directions for AI in ovarian cancer diagnosis and treatment.

Box 1 **AI integration in rare ovarian cancer subtypes.**

Currently, AI applications in these rare ovarian cancer subtypes are in their infancy. Preliminary studies, mostly radiomics-based, have demonstrated feasibility in subtype classification and outcome prediction. Additionally, some studies have started to explore specific subtypes, such as clear cell carcinoma, mucinous carcinoma, and undifferentiated carcinoma.

Ovarian clear cell carcinoma (OCCC):

OCCC is an uncommon subtype of epithelial ovarian cancer, often arising in an endometriosis setting and difficult to distinguish preoperatively. Emerging AI work has focused on risk stratification and imaging-based classification. Guo et al. (2023) applied ML algorithms to predict distant metastasis in OCCC. The RF model outperformed the others, achieving an accuracy of 0.792, sensitivity of 0.904, specificity of 0.759, and an AUC of 0.834. The Brier Score for the RF model’s calibration curve was 0.06256 (168). Similarly, Takeyama et al. (2024) applied MRI radiomic analysis to distinguish clear cell vs. endometrioid carcinomas, reporting ROC AUCs of 0.895, 0.910, and 0.956 for their MRI, radiomic, and combined models respectively (169). However, these studies are limited by small, single-institution cohorts and a lack of independent external validation. No AI models have explicitly incorporated molecular markers, for guiding OCCC prognosis or therapy.

Mucinous ovarian carcinoma (MOC):

AI research in mucinous ovarian carcinoma remains sparse. Available studies have explored imaging-based subtype classification. Yang et al. (2023) developed ultrasound-based radiomics models to predict the five major histological subtypes of epithelial ovarian cancer. For low-grade serous, endometrioid, and clear cell carcinomas, the models had AUCs below 0.80. For mucinous carcinoma, AUCs ranged from 0.80 to 0.89 (170). Separately, Hallberg et al. (2025) performed a large multi-omic genomic analysis of MOC, identifying recurrent alterations (e.g. SMARCA4, NF1) and showing that ovarian mucinous tumors have distinct mutation/methylation patterns from gastrointestinal mucinous tumors (171). Critically, distinguishing primary ovarian mucinous carcinoma from metastatic gastrointestinal mucinous tumors remains a significant challenge.

EOC:

EOCs are molecularly similar to uterine endometrial carcinomas, sharing similar alteration burdens and methylation profiles. EOCs accounts for ~10% of epithelial ovarian cancers and displays broad morphologic diversity that complicates diagnosis and grading. Takeyama et al. radiomics study treated endometrioid tumors as the comparator group and achieved AUC ≈ 0.956 for the combined model (169). In Yang et al.’s ultrasound radiomics analysis, the endometrioid carcinoma model yielded AUC below 0.80 (170). It is worth noting that no AI model has yet been reported for detecting microsatellite instability (MSI) or mismatch repair status in ovarian endometrioid carcinoma.

In summary, AI applications in these rare ovarian cancer subtypes are in their infancy. Future progress will likely depend on large-scale, multi-modal collaborations (imaging + pathology + genomics) to build robust AI models for these understudied subtypes.

Box 2 **Criteria and limitations of AI-ready EHR data.**

Key criteria for AI-worthy EHRs:

• Structured data: Machine-readable variables (e.g., labs, medications, vital signs).

• Standardized coding: Use of controlled vocabularies (ICD, SNOMED-CT, LOINC).

• Data completeness: Minimal missingness, especially for key clinical variables.

• Longitudinal records: Time-stamped data enabling dynamic modeling.

• Interoperability: Compatibility across systems (e.g., HL7 FHIR).

• Validated outcomes: Reliable ground truth labels (e.g., confirmed diagnoses, survival).

Major limitations of EHR data:

• Heterogeneity: Variability across institutions in data structure and coding.

• Missing data: Incomplete laboratory, symptom, and follow-up information.

• Bias: Unequal data quality across demographic groups.

• Unstructured data burden: Dependence on NLP for clinical notes.

• Privacy constraints: Restricted data sharing and limited external validation.

Practical recommendations:

• Harmonize coding systems and map local terminologies.

• Implement data quality control pipelines.

• Perform external validation across institutions.

• Conduct subgroup and fairness analyses.

• Ensure transparent reporting of preprocessing and data provenance.

The increasing use of AI in healthcare highlights the need for “AI-ready” EHR data. Frameworks such as CODE-EHR emphasize standardized coding, transparent phenotyping, and reproducible data definitions (172). The use of controlled vocabularies and interoperability standards such as HL7 FHIR further facilitates data integration across institutions. In addition, structured longitudinal data with time-stamped variables and validated outcomes are essential for dynamic modeling and robust prediction (173, 174). However, EHR data are often incomplete and heterogeneous, with missing values and inconsistent coding that can introduce bias and reduce generalizability; Unequal data representation across populations may further affect fairness in AI models. Therefore, the effectiveness of EHR-driven AI depends not only on algorithmic performance but critically on data quality, standardization, and fairness.

Box 3 **AI challenges and future directions in ovarian cancer.**

AI in ovarian cancer research shows great potential, but its clinical application is hindered by challenges like lack of transparency, interpretability, and integration of multi-modal data.

Challenges:

• Lack of transparency and interpretability of AI models.

• Heavy reliance on single-modal data, limiting the model's ability to handle tumor heterogeneity.

• Limited clinical validation and standardization across centers and technologies.

Future directions:

• Integrating multi-omics data with clinical and imaging information for improved prognostic modeling.

• Incorporating spatial biology and tumor-immune ecosystem analysis to enhance cancer diagnosis and therapy.

• Developing AI models with improved interpretability to support clinical decision-making and biological insights.

## 7 Conclusion

In summary, AI has demonstrated practical utility in ovarian cancer research, particularly in imaging-based triage, digital pathology, and clinical risk stratification. Integrating multimodal data has further improved precision. Collectively, these advances support the role of AI as a strong tool in ovarian cancer detection and management. Future progress will depend not on increasingly complex models, but on rigorous validation and clinically grounded design.

## Glossary

3D: Three-dimensional
ADNEX: Assessment of Different NEoplasias in the adnexa
ADNEX-AI: AI-enhanced ADNEX model
AE: Autoencoder
AI: Artificial Intelligence
ANN: Artificial Neural Network
AUC: Area Under the Curve
AUROC: Area Under the Receiver Operating Characteristic Curve
BCP: Borderline Cystic Pathology
BEOT: Borderline Epithelial Ovarian Tumor
CA125: Cancer Antigen 125
CDK4/6: Cyclin-Dependent Kinase 4/6
CEA: Carcinoembryonic Antigen
CI: Confidence Interval
CNN: Convolutional Neural Network
CRP: C-Reactive Protein
CT: Computed Tomography
CTCs: Circulating Tumor Cells
CyTOF: Cytometry by Time-of-Flight
C-index: Concordance Index
DCNN: Deep Convolutional Neural Network
ddPCR: Droplet Digital Polymerase Chain Reaction
DL: Deep Learning
DNN: Deep Neural Network
EHR: Electronic Health Record
EOC: Epithelial Ovarian Cancer
FC: Fully Connected (layer)
FDA: Food and Drug Administration
GEO: Gene Expression Omnibus
GRIPP: Guidance for Reporting Involvement of Patients and the Public
GNN: Graph Neural Network
H&E: Hematoxylin and Eosin
HCAI: Human-Centered Artificial Intelligence
HE4: Human Epididymis Protein 4
HGSOC: High-Grade Serous Ovarian Carcinoma
HMCAT: Hierarchical Multimodal Co-Attention Transformer
HRD: Homologous Recombination Deficiency
HRR: Homologous Recombination Repair
IHC: Immunohistochemistry
IOTA: International Ovarian Tumor Analysis
IOTA-ADNEX: International Ovarian Tumor Analysis Assessment of Different NEoplasias in the adneXa
KEGG: Kyoto Encyclopedia of Genes and Genomes
KNN: k-Nearest Neighbors
LASSO: Least Absolute Shrinkage and Selection Operator
LMR: Lymphocyte-to-Monocyte Ratio
LOH: Loss of Heterozygosity
LR: Logistic Regression
LST: Large-Scale Transitions
MIL: Multiple Instance Learning
ML: Machine Learning
MRI: Magnetic Resonance Imaging
MTIL: Malignant transcriptional program that robustly marks the presence of TILs
MMD-VAE: Maximum Mean Discrepancy Variational Autoencoder
MOC: Mucinous Ovarian Carcinoma
NLP: Natural Language Processing
NLR: Neutrophil-to-Lymphocyte Ratio
OCCC: Ovarian Clear Cell Carcinoma
OCDPI: Ovarian Cancer Digital Pathology Index
PARP: Poly (ADP-ribose) Polymerase
PCORI: Patient-Centered Outcomes Research Institute
PET: Positron Emission Tomography
PFS: Progression-Free Survival
PLR: Platelet-to-Lymphocyte Ratio
PRO: Patient-Reported Outcomes
ResNet-FPN: Residual Network with Feature Pyramid Network
RF: Random Forest
RNN: Recurrent Neural Network
ROMA: Risk of Ovarian Malignancy Algorithm
SBOTs: Serous Borderline Ovarian Tumors
SMOTs: Seromucinous Ovarian Tumors
SVM: Support Vector Machine
STICs: Serous Tubal Intraepithelial Carcinomas
TAI: Telomeric Allelic Imbalance
TCGA: The Cancer Genome Atlas
TP53: Tumor Protein p53
UMAP: Uniform Manifold Approximation and Projection
UMLS: Unified Medical Language System
US: Ultrasound
VAE: Variational Autoencoder
VTE: Venous Thromboembolism
ViT: Vision Transformer
WSI: Whole-Slide Image
XGBoost: Extreme Gradient Boosting
XAI: Explainable Artificial Intelligence
Bias: Systematic error introduced during data collection, labeling, model development, or evaluation that can distort predictions and reduce fairness or reliability across patient groups
Calibration: The agreement between predicted probabilities and observed outcomes. A well-calibrated model produces risk estimates that accurately reflect real-world event likelihood
Cell-type deconvolution: A computational approach used to estimate the proportions or spatial distributions of different cell types within complex tissue data, particularly in spatial omics datasets
Clinical translation: The process of moving an AI model from research development into real-world clinical use through validation, workflow integration, regulatory review, and demonstration of clinical utility
Clinical utility: The extent to which a model meaningfully improves clinical decision-making or patient outcomes, beyond showing good technical performance alone
Decision-curve analysis (DCA): A method for evaluating the clinical usefulness of predictive models by quantifying net benefit across a range of decision thresholds
Digital pathology: The use of digitized histopathological slides combined with computational analysis, often powered by AI, to assist diagnosis, prognosis, biomarker discovery, and treatment stratification
Domain shift: Differences between training data and real-world clinical data that can lead to decreased model performance when deployed in new settings
Early/Intermediate/Late fusion: Strategies for integrating multimodal data at different stages of model development (input-level, feature-level, or decision-level, respectively)
Electronic health records (EHR): Digitally stored clinical information, including demographics, diagnoses, laboratory results, treatment history, medications, and clinical notes, used for predictive modeling and real-world evidence generation
Explainable AI (XAI): Methods and techniques that make AI model outputs interpretable by highlighting important features, regions, or decision pathways
External validation: Evaluation of a model on independent datasets from different institutions or populations to assess generalizability and real-world performance
Feature space: A representation in which each sample is described by a set of features or variables, enabling models to learn patterns across high-dimensional data
Federated learning (FL): A distributed machine learning approach in which models are trained across multiple institutions without sharing raw data, preserving privacy while enabling collaboration
Foundation model: A large pre-trained model that learns generalizable representations from broad datasets and can be adapted to specific downstream tasks
Generalizability: The ability of a model to maintain performance across different datasets, institutions, or populations beyond the training environment
Human-AI collaboration: A framework in which AI supports, rather than replaces, human experts, enhancing decision-making while maintaining clinical oversight
Interpretability: The extent to which a human can understand how or why an AI model produces a given prediction
Latent representation: A compressed internal representation learned by a model that captures essential structure in the data, often used for multimodal integration
Ligand-receptor interaction: A biological signaling event in which a ligand released by one cell binds to a receptor on another, used to infer cell-cell communication
Longitudinal data: Data collected repeatedly over time from the same individual, enabling modeling of disease progression and treatment response
Multimodal AI: AI approaches that integrate multiple data modalities to improve prediction, interpretation, and decision support
Multimodal data: Data derived from multiple sources such as imaging, genomics, pathology, and clinical records, providing complementary information
Pathomics: Quantitative analysis of pathology images to extract morphological and spatial features associated with biological or clinical outcomes
Radiomics: Extraction of large numbers of quantitative features from medical images to characterize tumor phenotype and heterogeneity
Spatial omics: Technologies that measure gene or protein expression while preserving spatial context within tissue
Spatial proteomics: Spatially resolved measurement of protein expression within tissue sections
Spatial transcriptomics: Methods that measure RNA expression while preserving spatial location within tissue
Subclone: A genetically or transcriptionally distinct subgroup of tumor cells within a tumor
Tumor microenvironment (TME): The cellular and molecular environment surrounding tumor cells, including immune cells, stromal components, and signaling factors
Whole-slide image (WSI): A high-resolution digital scan of a complete histopathology slide enabling computational analysis.
